# *In vivo* immune interactions of multipotent stromal cells underlie their long-lasting pain-relieving effect

**DOI:** 10.1038/s41598-017-10251-y

**Published:** 2017-08-31

**Authors:** Wei Guo, Satoshi Imai, Jia-Le Yang, Shiping Zou, Mineo Watanabe, Yu-Xia Chu, Zaid Mohammad, Huakun Xu, Kamal D. Moudgil, Feng Wei, Ronald Dubner, Ke Ren

**Affiliations:** 1 Department of Neural and Pain Sciences, School of Dentistry, & Program in Neuroscience, University of Maryland, Baltimore, MD 21201 USA; 2 Division of Biomaterials and Tissue Engineering, School of Dentistry, University of Maryland, Baltimore, MD 21201 USA; 3 Department of Microbiology & Immunology, University of Maryland, Baltimore, MD 21201 USA; 4 Present Address: Department of Clinical Pharmacology and Therapeutics, Kyoto University Hospital, 54 Shogoin Kawahara-cho, Sakyo-ku, Kyoto, 606-8507 Japan; 5 Present Address: Department of Orthodontics and Craniofacial Developmental Biology, Hiroshima University, Graduate School of Biomedical Sciences, 1-2-3 Kasumi, Minami-ku, Hiroshima, 734-8553 Japan; 6 Present Address: Department of Integrative Medicine and Neurobiology, School of Basic Medical Sciences, Fudan University, Shanghai, China

## Abstract

Systemic infusion of bone marrow stromal cells (BMSCs), a major type of multipotent stromal cells, produces pain relief (antihyperalgesia) that lasts for months. However, studies have shown that the majority of BMSCs are trapped in the lungs immediately after intravenous infusion and their survival time in the host is inconsistent with their lengthy antihyperalgesia. Here we show that long-lasting antihyperalgesia produced by BMSCs required their chemotactic factors such as CCL4 and CCR2, the integrations with the monocytes/macrophages population, and BMSC-induced monocyte CXCL1. The activation of central mu-opioid receptors related to CXCL1-CXCR2 signaling plays an important role in BMSC-produced antihyperalgesia. Our findings suggest that the maintenance of antihypergesia can be achieved by immune regulation without actual engraftment of BMSCs. In the capacity of therapeutic use of BMSCs other than structural repair and replacement, more attention should be directed to their role as immune modulators and subsequent alterations in the immune system.

## Introduction

Multipotent stromal cells have shown their therapeutic potential in a variety of clinical conditions^1–3^. Transplantation of bone marrow stromal cells (BMSCs), a major type of multipotent stromal cells, produces pain-relief or antihyperalgesia that lasts up to months and apparently involves activation of endogenous opioids in preclinical pain models^4–8^. Clinical studies also show promising long-term pain relief with multipotent stromal cells^9, 10^.

Considering the long-lasting beneficial effects of MSCs, there is a paradox. MSCs are often administered systemically. However, the majority of MSCs are trapped in the lungs immediately after intravenous infusion^11–13^. Only a very small percentage (<1%) of systemic MSCs can migrate to the injured brain/spinal site^12, 14–16^. Even direct arterial infusion that bypasses the pulmonary first-pass effect only leads to a transient recruitment of the cells to the brain^17^. Further, systemic MSCs only stay in the body for a matter of days to a few weeks^12, 13, 18^. MSCs survived better after intrathecal delivery. A portion of intrathecally injected MSCs migrated to the dorsal root ganglion and survived there for up to 84 days^4^. Nevertheless, about 50% of the survived cells were lost in about 14 days, while the antihyperalgesia of MSCs remained at the same level for at least 38 days.

Recent MSC medicine appreciate that there are sophisticated interactions between implanted cells and the host immune system^19, 20^. Intravenously infused MSCs would first encounter circulating immune cells and embolized cells in the lungs also interact with the host^13, 21, 22^. MSCs express a variety of immune mediators and receptors^23, 24^. The interactions between the immune and nervous system affect pain^25^. Thus, infused MSCs may interact with host immune cells, followed by release of immune mediators, leading to activation of the endogenous analgesic system and long-lasting pain relief. We have conducted a series of experiments to test this hypothesis.

## Results

### BMSC induced upregulation of opioid receptors

We have used a model of chronic orofacial pain with ligation injury of the masseter muscle tendon (TL) to assess BMSC-produced antihyperalgesia^5^. Notably, the pain-attenuating effect of BMSCs was consistently observed in other persistent pain models in both males and females with multiple measures of nociception, including thermal and mechanical pain sensitivity and pain-related conditioned place avoidance^26^. We showed previously that the BMSC-produced antihyperalgesia was attenuated, or pain hypersensitivity rekindled, by naloxone, an opioid receptors antagonist^5^; and that N-methyl-D-aspartate receptor-mediated trigeminal nociceptive neuronal hyperexcitability was attenuated by BMSCs, an effect also reversed by naloxone^26^. As naloxone may act as an inverse agonist to block opioid receptor constitutive activity, leading to increased pain^27^, we further verified this effect with a neutral opioid receptor antagonist 6-β-naltrexol. Similarly, the BMSC-produced antihyperalgesia was attenuated by 6-β-naltrexol (Supplementary Fig. 1a,b). These results suggest that the antihyperalgesia by BMSCs involves the endogenous opioid system.

**Figure 1:**
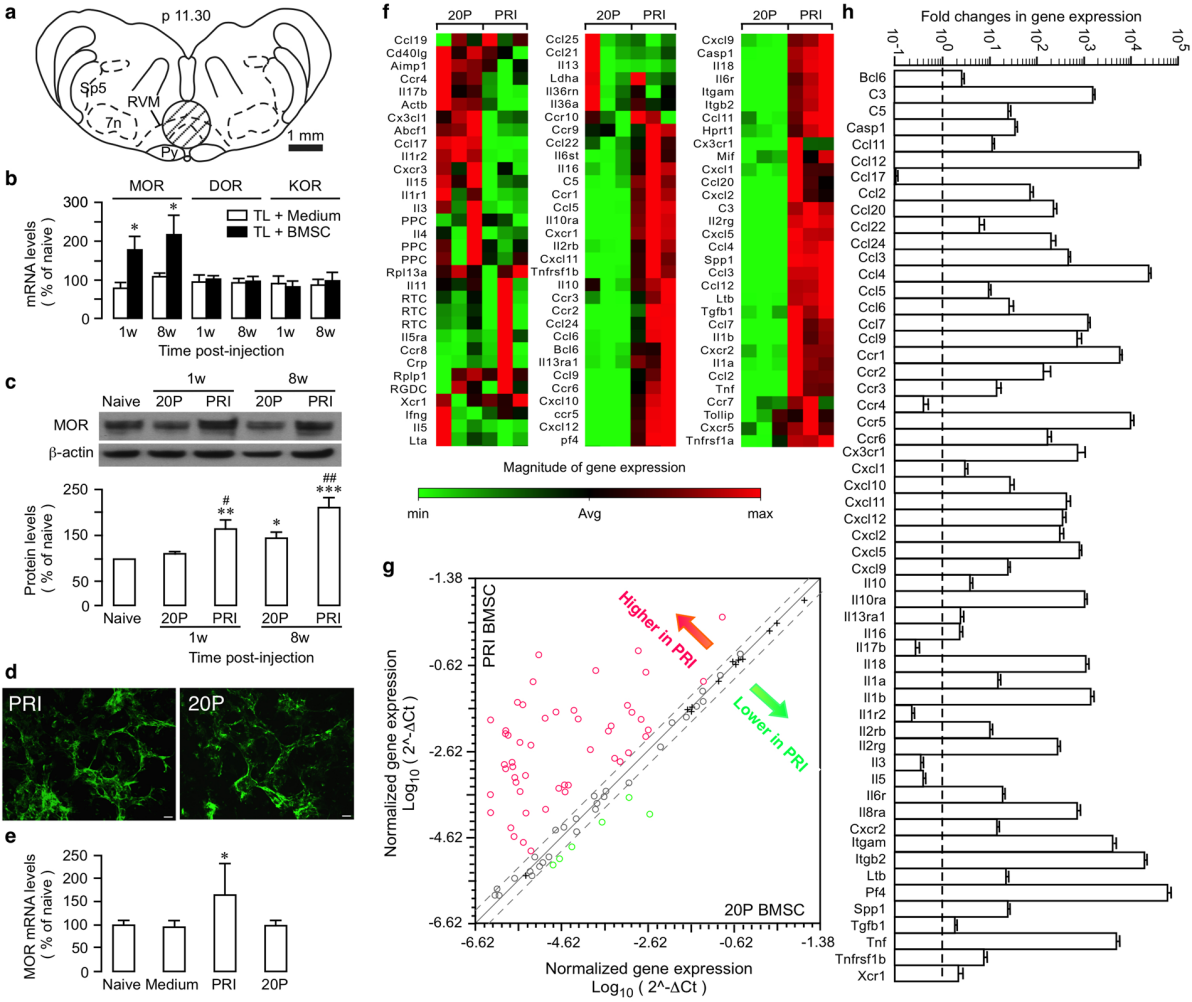
BMSC-induced selective upregulation of MOR and differential gene expression profiles between PRI and 20P-BMSCs. (**a**) The transverse section of the brainstem at 11.30 mm posterior to the Bregma illustrating the site where rostral ventromedial medulla (RVM) tissues were punched out for analysis (circle). py, pyramidal tract; 7n, facial nucleus; sp5, spinal trigeminal complex. (**b**) Bar graph shows relative levels of opioid receptor mRNAs in RVM. *p < 0.05 vs. medium. MOR, DOR, KOR: mu-, delta-, and kappa-opioid receptors, respectively. N = 3/group. (**c**) TL rats received primary (PRI) or 20-passage (20 P) BMSCs (20P-BMSC do not produce antihyperalgesia and was used as a control) at 7d post injury. β-actin was a loading control. *p < 0.05, **p < 0.01, ***p < 0.001, vs. Naïve; ^#^p < 0.05, ^##^p < 0.01, vs. respective 20 P. n = 6/group. Cropped gel images are shown. See Supplementary Figure 9 for full-length blots. (**d**) Immunostaining against CD90 in PRI and 20 P BMSCs. Scale = 10 μm. (**e**) The treatment with PRI-, but not 20P-BMSCs, upregulated MOR gene expression in RVM. *p < 0.05, vs. naïve, medium and 20 P, n = 3. For (**b**,**c**,**e**), statistical comparisons were made by One-way ANOVA followed by post-hoc comparisons with Bonferroni corrections. (**f**) Heat map of gene-expression analysis. Total RNAs from PRI- and 20P-BMSCs were characterized using The Rat Inflammatory Cytokines & Receptors RT^2^ Profiler^TM^ PCR Array. Each column shows a separate sample. (**g**) The relative expression levels of each gene (open circles) in the PRI and 20 P samples are plotted against each other. *Red*, genes shown higher expression levels in PRI-BMSCs (>2-fold); *Green*, genes expressed lower (>2-fold) in PRI-cells and *Black*, no change (≤2-fold). The two dashed lines indicate the 2-fold changes from the middle diagonal line. Crosses represent control genes. (**h**) Bar graph illustrating fold changes in gene expression between PRI and 20P-BMSCs. Dashed line represents equal expression between PRI- and 20P-BMSCs.

Since RNAi of μ-opioid receptors (MOR) in the rostral ventromedial medulla (RVM), a major opioid-containing brainstem site for pain modulation, attenuated BMSC-produced antihyperalgesia^5^, we first examined whether there was a long-term effect of BMSCs on opioid receptor expression in the RVM (Fig. 1a). At 1w and 8w after infusion of primary BMSC, RT-qPCR showed that MOR, but not δ- and κ-opioid receptor mRNAs was upregulated (Fig. 1b) and western blot confirmed upregulation of MOR proteins in RVM (Fig. 1c). We have found that BMSCs after multiple passages (≥20 P) lost their antihyperalgesic property^5^ and can be used as a control. 20P-BMSCs did not affect MOR expression at 1w but only slightly increased MOR at 8w after infusion (Fig. 1c). Compared to 20P-BMSC-treated rats, the MOR protein levels were significantly higher at both 1 and 8 w after treatment with primary BMSCs (Fig. 1c). Consistently, increased immunostaining against MOR in RVM was observed (Supplementary Fig. 1c–f). This phenomenon was not limited to tissue injury pain models. In rats with L5 spinal nerve ligation injury, a model of neuropathic pain, infusion of human BMSCs selectively upregulated MOR mRNA in RVM (Supplementary Fig. 1h). These observations indicate that BMSC-produced antihyperalgesia involves long-lasting activation of the endogenous opioid system.

### Differential gene expression in primary and 20P-BMSCs

The lasting upregulation of MOR and antihyperalgesia apparently contradicts the kinetics of infused BMSCs in the body. Most intravenously infused MSCs are trapped in the lungs and removed from the system within a few weeks^12, 13^. Utilizing BMSCs isolated from GFP transgenic S-D rats [SD-Tg(GFP)Bal]^28^, we have verified this phenomenon. BMSCs were infused into the rat at 7d after TL. GFP-positive BMSCs were accumulated in the lungs at 6 h and 1d after infusion and very few GFP cells were observed at 7d after infusion (Supplementary Fig. 2). Few GFP cells were seen at the injured site and none was observed in the brainstem. Considering long-lasting effect of BMSCs on MOR and persistent pain, we reasoned that mechanisms other than a direct effect or engraftment of BMSCs underlie their antihyperalgesia.

Previous studies indicate that primary, but not 20P-BMSCs, produced pain relief^5^, suggesting altered BMSC genotype during culturing. Primary and 20P-BMSCs exhibited similar staining intensity for CD90.1 (Fig. 1d), indicating that BMSC markers maintained their expression in high-passage BMSCs^29^. However, at 8w after BMSC infusion, primary, but not 20P-BMSCs, upregulated MOR gene expression in RVM (Fig. 1e), consistent with a lack of antihyperalgesic effect by 20P-BMSCs^5^. Taking advantage of this phenomenon, we compared gene expression of primary and 20P-BMSCs. Utilizing the Inflammatory Cytokines & Receptors PCR Array, we observed differential expression profiles of major cytokines/chemokines and receptors between the primary and 20P-BMSCs (Fig. 1f–h). Among the 84 genes examined, 50 in primary BMSCs were expressed at a higher level in all 3 samples (≥2-fold) and 26 of them showed greater than 100-fold differences over the 20P-cells. Six genes expressed lower (>2-fold) in primary BMSCs (Fig. 1h). Genes for ribosomal proteins (*Rpl13a* and *Rplp1*), lactate dehydrogenase A and housekeeping protein β-actin (*Actb*) do not show differential expressions, suggesting normal biochemical activity remained in 20P-BMSCs. Control genes did not show greater than 2-fold differences between primary and 20P-BMSCs (Fig. 1g, crosses). Higher expression of some genes was further verified with RT-qPCR (Supplementary Fig. 3)

A repertoire of genes for immune mediators and receptors exhibited higher levels of expression in primary BMSCs compared to 20P-BMSCs (Table 1 ^4, 22, 30–44^), including genes for chemotaxis-inducing chemokines and their receptors (*Cxcl4, Ccl2, Ccl3, Ccl4, Cxcl12 (Sdf-1), Ccr2, Ccr5*), anti-inflammatory cytokines and receptors (*Tgfb1*, *Il10, Il10-ra, Il13RA1), proinflammatory cytokines and receptors (Tnf, Il1b, Il18, Tnfrsf1b)*, and components of the complement pathways (*C3, C5*). Interestingly, genes for CXCR2 and its ligands *(Cxcl1, Cxcl2, Cxcl5, Cxcr2)* that are involved in opioid release from immune cells^30^ showed higher expression in primary BMSCs. There was simultaneous higher mRNA expression of some receptors and their ligands in primary BMSCs (e.g., *Ccl3*/*4/5-Ccr5*, *Cxcl1/2-Cxcr2, Ccl5-Ccr1*, *Il10-Il10ra*, *Tnf-Tnfrsf1b*, *Cxcl1/2/5-Cxcr2*), suggesting reciprocal interactions with the host immune cells and autocrine signaling of BMSCs. TGFβ1, which was a critical mediator of BMSCs’ antihyperalgesia after intrathecal administration^4^, expressed higher in primary BMSCs (Fig. 1h, Supplementary Fig. 3). While higher expression of the anti-inflammatory cytokines and receptors is consistent with antihypergesia, genes of proinflammatory cytokines and complement components may contribute to the effect of BMSCs by promoting chemotactive interactions with immune cells^21^.

### BMSC-derived Chemokines as mediators of antihyperalgesia

**Figure 2:**
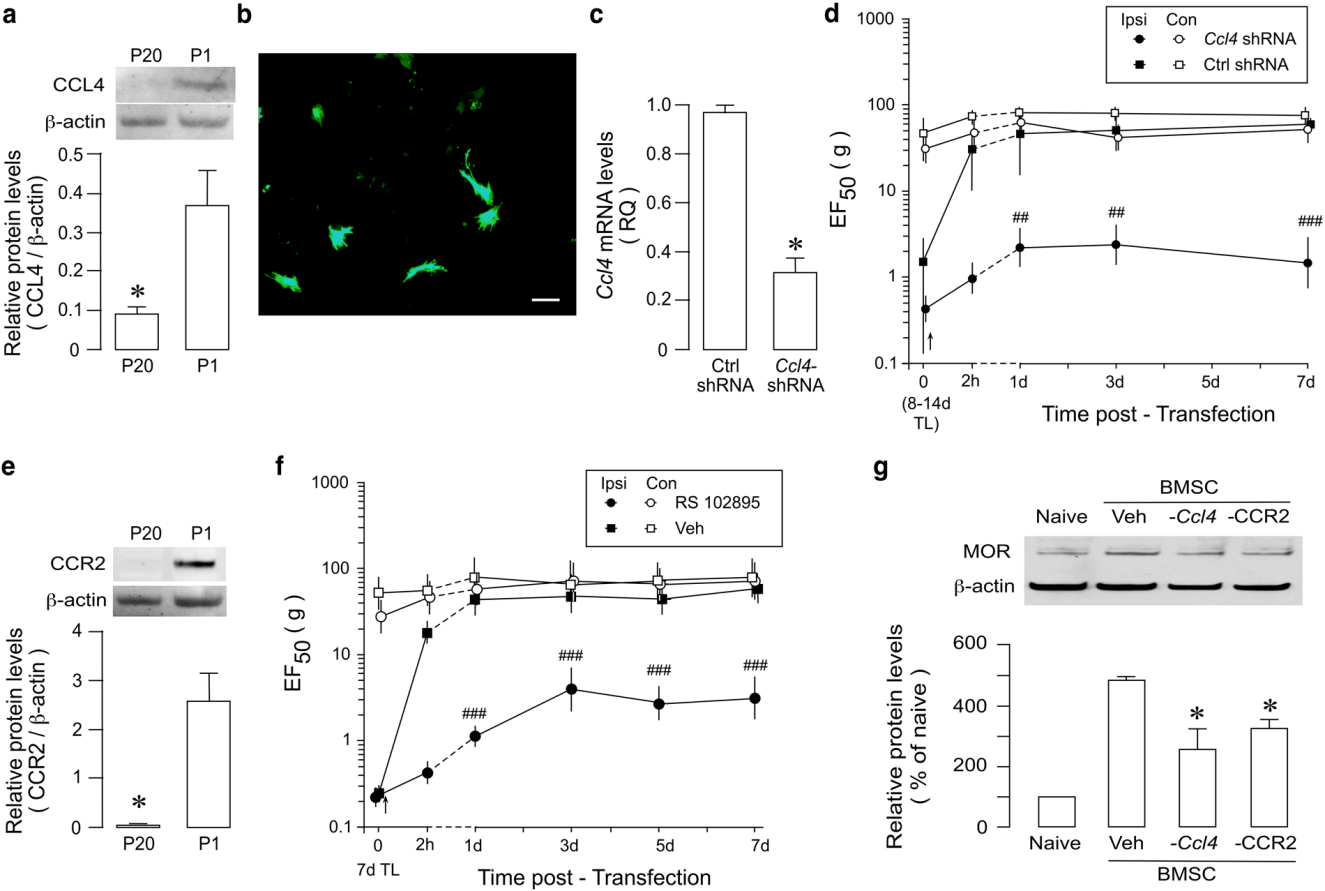
Attenuation of BMSC-induced antihyperalgesia after subtraction of *Ccl4* and blocking CCR2. (**a**) CCL4 protein levels were reduced in P20 BMSCs. *p < 0.05, vs. P1 BMSCs, n = 4. (**b**) Primary BMSCs were transducted with *Ccl4*-shRNA plasmids containing GFP. Immunofluorescence staining of BMSC with GFP confirmed successful gene transfer. Scale = 10 μm. (**c**) *Ccl4* mRNA expression was reduced in BMSCs transduced with *Ccl4* shRNA lentivirus compared to Ctrl shRNA-treated cells (n = 3, RT-qPCR). *p < 0.05, vs. ctrl shRNA. (**d**) Attenuation of BMSC-induced antihyperalgesia by pretreatment of BMSCs with RNAi of *Ccl4*. Compared to Ctrl shRNA, *Ccl4* shRNA pretreatment attenuated BMSC-produced antihyperalgesia in TL rats, as indicated by a reduction in EF_50_. Dashed lines connect data points not shown with the scale of the abscissa. Ipsi, ipsilateral to injury; Con, contralateral non-injured. (**e**) CCR2 protein levels were reduced in P20 BMSCs. *p < 0.05, vs. P1 BMSCs, n = 4. (**f**) Pretreatment of BMSC with RS 102895 (10 μM for 24 h), a CCR2b antagonist attenuated BMSC-produced antihyperalgesia. ^##^p < 0.01, ^###^p < 0.001; vs. Ctrl shRNA (C) and vs. Veh (D). (**g**) Pretreatment of BMSC with shRNA against *Ccl4* (-*Ccl4*) or CCR2b antagonist RS 102895 (-CCR2) reduced BMSC-induced MOR upregulation in RVM in TL rats. *p < 0.05, vs. veh. Error bars in (**a**,**c**,**e**, and **g**) are S.E.M. and in (**d** and **f**) (n = 6–8) are 95% confidence intervals. Statistics: (**a**,**c**,**e**) Unpaired, Two-tailed Student’s *t*-test; (**d**,**f**) Two-way ANOVA followed by post-hoc comparisons with Bonferroni corrections; (**g**) One-way ANOVA followed by post-hoc comparisons with Bonferroni corrections. Cropped gel images are shown in 2a, e.g. See Supplementary Figure 9 for full-length blots.

Differential gene expressions between antihyperalgesic primary and ineffective 20P-BMSCs suggest a role of BMSC-derived chemokines in BMSC-produced antihyperalgesia. We next chose to evaluate a role of CCL4 since it’s mRNA was not detectable in 2 of 3 20P-BMSC samples (*Ct* over the cut-off in one) and its expression showed the second highest fold change over 20P-BMSCs (Fig. 1h, Table 1). Western blot confirmed very low level of CCL4 in 20P-BMSCs, compared to primary BMSCs (Fig. 2a). We then employed a subtraction strategy to assess the role of CCL4 in BMSC-produced antihyperalgesia. Before transplantation, primary BMSCs were transduced with *Ccl4* shRNA lentivirus. Figure 2b,c show the successful transduction of BMSCs and knock-down of *Ccl4*. Mechanical nociception of the rat was assessed with von Frey filaments^45^. EF_50_ (Effective Force50), the derived von Frey filament force (g) that produces a 50% response, was used as a measure of mechanical sensitivity^5^. In TL rats receiving infusion of transduced BMSCs, BMSC-induced increase in EF_50_ was significantly reduced, compared to that receiving control shRNA (Fig. 2d), and the increased MOR expression in RVM was also significantly reduced (Fig. 2g). These results indicate that BMSC-derived CCL4 contributed to their antihyperalgesic effect.

**Table 1:** Summary of highly expressed genes in primary- vs. 20P-BMSCs.

Genes	Fold change	Note
***Chemotaxis/migration of immune cells***		
*Pf4/Cxcl4*	60633 ± 8047	Platelet factor 4 (PF4), promoting coagulation, chemotactic for neutrophils and fibroblasts. Role in monocyte activation^31^
*Ccl4*	23470 ± 492	Macrophage inflammatory protein 1β (MIP-1β), chemotactic for monocytes^32^ and T-cell^33^
*Itgb2*	18959 ± 1385	Integrin beta-2 (CD18), Leukocyte adhesion
*Ccl12*	14047 ± 596	Monocyte chemotactic protein-5 (MCP-5), attracts eosinophils, monocytes and lymphocytes
*Ccr5*	9960 ± 1401	Receptor for CCL4/CCL5. CCR5 oligomerizes with CCR2 and CXCR4^34^.
*Ccr1*	5939 ± 568	Receptor for CCL5, recruitment of effector immune cells.
*Itgam*	4270 ± 332	CD11b, Macrophage-1 antigen, regulating leukocyte adhesion and migration.
*Ccl3*	465 ± 12	Macrophage inflammatory protein 1α (MIP-1α), Interact with CCL4^35^. Attracts macrophages, monocytes and neutrophils.
*Cxcl12*	366 ± 45	Stromal cell-derived factor 1 (SDF-1), chemotactic for lymphocytes^36^; Binds CXCR4, important for homing of MSCs to the injured site^37^.
*Ccr2*	149 ± 48	CD192, Receptor for CCL2 (MCP-1), Important for MSC homing^38, 39^, and monocyte recruitment^40^
*Ccl2*	78 ± 7	Monocyte chemotactic protein-1 (MCP-1), mediating monocyte chemotaxis signalling through CCR2.
*Ccl11*	12 ± 1	CCL11 heterodimerizes with CCL2^41^
*Ccl5*	10 ± 1	RANTES, CCR5 ligand, role in peripheral opioid expression^42^.
***Antiinflammatory cytokines/receptors***		
*Il10ra*	1034 ± 78	IL-10 receptor subunit alpha.
*Il10*	4 ± 0.4	Role in BMSC’s anti-sepsis effect^22^.
*IL-13RA1*	3 ± 0.2	IL-13 receptor, alpha 1.
*Tgfb* (TGFβ)	2 ± 0.1	Role in BMSC’s antihyperalgesia^4^.
***Proinflammatory cytokines/receptors***		
*Tnf*	5019 ± 534	Also stimulate MOR gene expression in neurons^43^.
*Il1b*	1438 ± 149	Important mediator of inflammatory response.
*Il18*	1104 ± 61	Inducing interferon-gamma, which leads to activation of macrophage.
*Tnfrsf1b*	8 ± 1	TNFR2, CD120b, receptor for TNF.
*Casp1*	36 ± 2	Caspase 1, Cleavage of precursors of the inflammatory cytokines IL-1β and IL-18.
*C3*	1501 ± 74	Complement component 3, related to BMSC activity^21^.
*C5*	26 ± 2	Complement component 5, related to BMSC activity^21^.
***Regulation of opioids***		
*Cxcl5*	793 ± 29	LIX, CXCR2 ligand, LPS-induced chemokine, neutrophil-activating peptide 78 (ENA-78).
*Cxcl2*	316 ± 59	MIP-2α, CXCR2 ligand, chemotactic for polymorphonuclear leukocytes.
*Cxcr2*/*Il8rb*	15 ± 1	Receptor for CXCL1/CXCL2, involved in opioid release from polymorphonuclear leukocytes^30^.
*Cxcl1*	3 ± 0.3	CXCR2 ligand; Related to opioid release from polymorphonuclear leukocytes^44^.

We then examined the role CCL2-CCR2 signaling, the most studied chemoattractant for monocytes, T cells and other immune cells^46^. Both CCL2 and CCR2 genes showed higher expression in primary BMSCs (Fig. 1h, Supplementary Fig. 3). In contrast to primary BMSCs, CCR2 proteins in 20P-BMSCs was almost undetectable (Fig. 2e). In the next experiment, primary BMSCs were pretreated with a CCR2 receptor antagonist RS102895 (10 μM for 24 h) before infusion. In TL rats receiving infusion of RS102895-treated cells, BMSC-induced increase in EF_50_ was significantly decreased (Fig. 2f); and the increased MOR expression in RVM was also significantly reduced (Fig. 2g). Since CCR2 has been shown important for MSC homing^38, 39^, we examined whether blocking CXCL12 (SDF-1)-CXCR4 signaling, key to migration of BMSCs to the injured site^4^, led to a reduction of antihyperalgesia. TL rats were pretreated with AMD3100 (plerixafor, 10 mg/kg/day, i.p. for 4 days), an antagonist of CXCL12 receptor CXCR4. AMD3100 did not affect BMSC-produced antihyperalgesia (Supplementary Fig. 4). These results suggest that pain attenuation after systemic BMSCs involves chemokine interactions with immune cells, but not necessarily involving homing. However, intrathecal BMSCs may escape the lung trapping and CXCR4 signaling appears important for the antihyperalgesia induced by intrathecal BMSCs^4^.

### The role of monocytes/macrophages in pain-relieving effect of BMSCs

**Figure 3:**
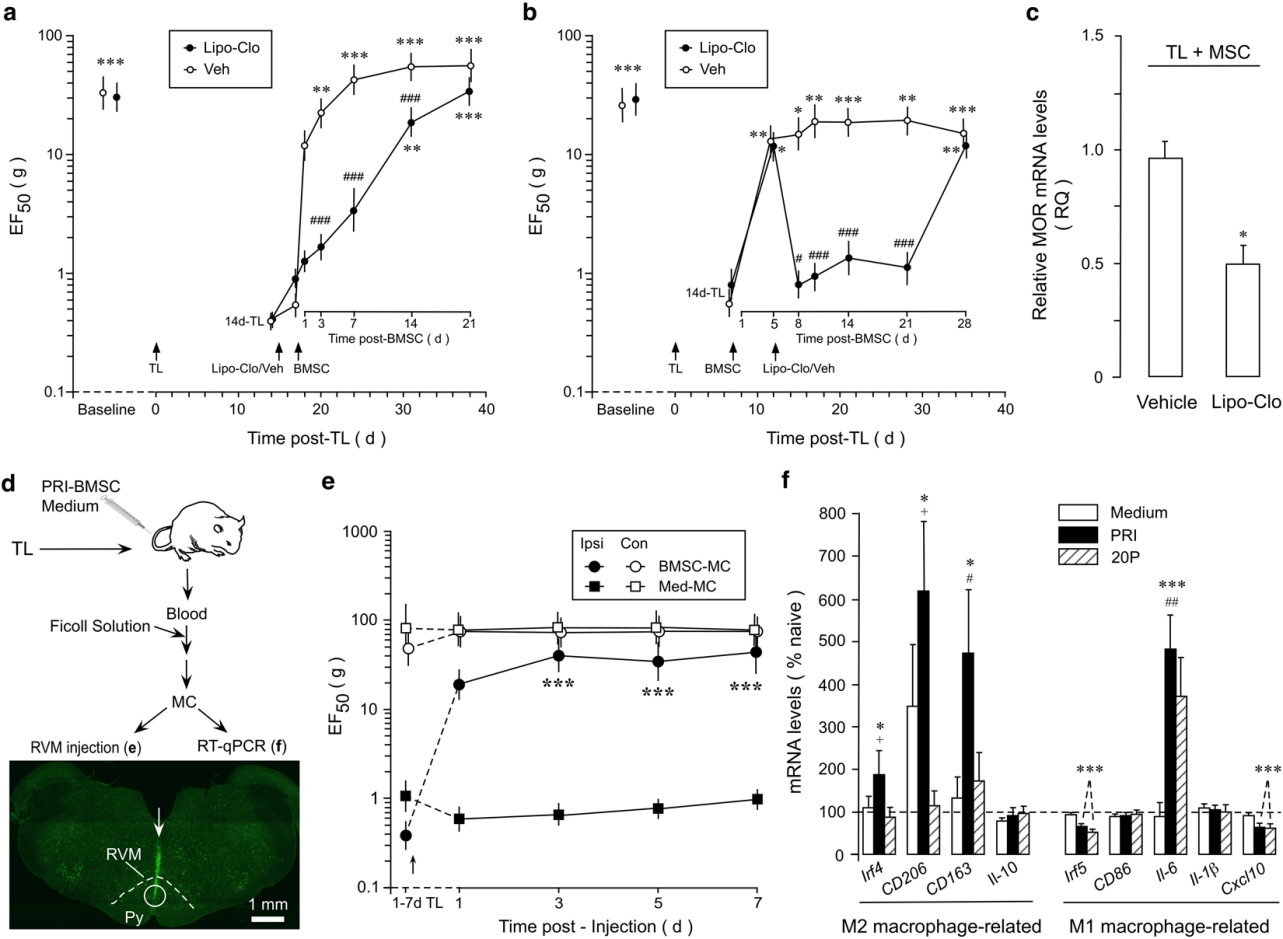
The role of monocyte/macrophage in BMSC’s antihyperalgesia. Liposome-encapsulated clodronate (Lipo-Clo) was injected i.p. 3 times in three days (10 mg/2 ml/injection/rat). (**a**,**b**) Lipo-Clo treatment attenuated BMSC-produced antihyperalgesia (**a**, pretreatment, **b**, post-treatment). Compared to Veh-treated rats, Lipo-Clo treatment led to a reduction in EF_50_. Veh: Phosphate buffered saline-filled liposome. Error bars are 95% confidence intervals. *p < 0.05, **p < 0.01, ***p < 0.001, vs. 14d-TL; #p < 0.01, ###p < 0.001, vs. veh. n = 5-9/Group. (**c**) RVM MOR mRNA levels were reduced in Lipo-Clo-treated rats (Sample taken at 7d post-Lipo-Clo from BMSC-treated rats). P < 0.05, n = 3. (**d**) Flow chart of the experiments shown in (**e** and **f**). Bottom image shows the injection site in RVM. Coronal brainstem sections were stained with green fluorescent Nissl stain. Arrow indicates the injection needle track and circle shows the site of injection. (**e**) Injection of MC (Arrow, 10,000 cells/0.5 μl) from BMSC-treated TL animals into the RVM attenuated behavioural hyperalgesia in TL rats, as compared to MC from medium-treated TL rats. ***p < 0.001 vs. Med-MC, n = 6/Group. (**f**) BMSC induced changes in monocyte phenotype. RT-qPCR was performed on peripheral blood monocytes isolated from TL rats treated with primary or 20P-BMSC or culture medium. Note that four M2 macrophage related markers were upregulated in primary BMSC-treated rats. *p < 0.05, ***p < 0.001, vs. naive; ^#^p < 0.05, ^##^p < 0.01, vs. medium; ^+^p < 0.05, vs. 20 P, n = 3. Statistics: (**a**,**b**,**e**) Two-way ANOVA followed by post-hoc comparisons with Bonferroni corrections; (**c**) Unpaired, Two-tailed Student’s *t*-test; (**f**) One-way ANOVA followed by post-hoc comparisons with Bonferroni corrections.

Our observations led to a hypothesis that host immune cells relay BMSCs’ input through chemotactic interactions to produce long-lasting antihyperalgesia. Monocytes/macrophages have been shown to mediate the BMSC-produced anti-sepsis^22^. Depletion of macrophages attenuated opioid-induced analgesia^47^. To evaluate a role of monocytes/macrophages, we employed the liposome-encapsulated clodronate (Lipo-Clo) procedure to deplete monocytes/macrophages^48–50^. Liposome-encapsulated clodronate was injected i.p. 3 times in three days (10 mg/2 ml/injection/rat). After the injections, peritoneal cells were collected and labeled with macrophage marker CD11b. The reduced peritoneal macrophage expression was verified by flow cytometry and ED1-immunostaining of spleen sections (Supplementary Fig. 5a,b). The Lipo-Clo treatment attenuated BMSC-produced antihyperalgesia. In rats pretreated with vehicle of Lipo-Clo, the EF_50_s were significantly increased from 3 d post-BMSCs compared to 14d-TL, indicating antihyperalgesia (Fig. 3a). However, with Lipo-Clo pretreatment, the EF_50_s were not significantly increased until 14-d after BMSCs when they were still lower than that of veh-treated rats (p < 0.001). When stable antihyperalgesia had been established, post-treatment with Lipo-Clo at 5d after BMSC infusion rekindled hyperalgesia between 8-21 d after BMSC (Fig. 3b). The MOR mRNAs were also significantly reduced in Lipo-Clo-treated rats (Fig. 3c).

To further confirm a role of monocytes/macrophage population in BMSCs’ antihyperalgesia, we isolated peripheral blood monocytes (MC) from TL rats that received either BMSCs or culture medium treatment (Fig. 3d). Flow cytometry showed that 30.9% of gated population was positive for monocyte marker CD11b (Supplementary Fig. 6a). This population was further purified by FACS. The isolated monocytes show characteristic CD11b and CD68 immunostaining (Supplementary Fig. 6b). Direct intra-RVM injection of monocytes from BMSC-treated animals attenuated hyperalgesia, as compared to monocytes from medium-treated rats (Fig. 3d,e). The two experiments involving manipulation of monocytes/macrophages provide convergent support for the involvement of monocytes/macrophages in BMSC-induced antihyperalgesia. Interestingly, some markers of M2 macrophages showed increased mRNA expression in monocytes from primary BMSC-treated rats, including *Irf4*, *CD206* and *CD163* (Fig. 3f, left). *Irf5* and *Cxcl10*, genes related to M1 macrophage showed decreased expression after BMSCs and *Il-6* gene expression was increased in both PRI-BMSC-treated rats (Fig. 3f, right). These results suggest that BMSCs promote polarization of macrophages toward an anti-inflammatory phenotype.

### Monocytes-derived CXCL1 contributes to antihyperalgesia

**Figure 4:**
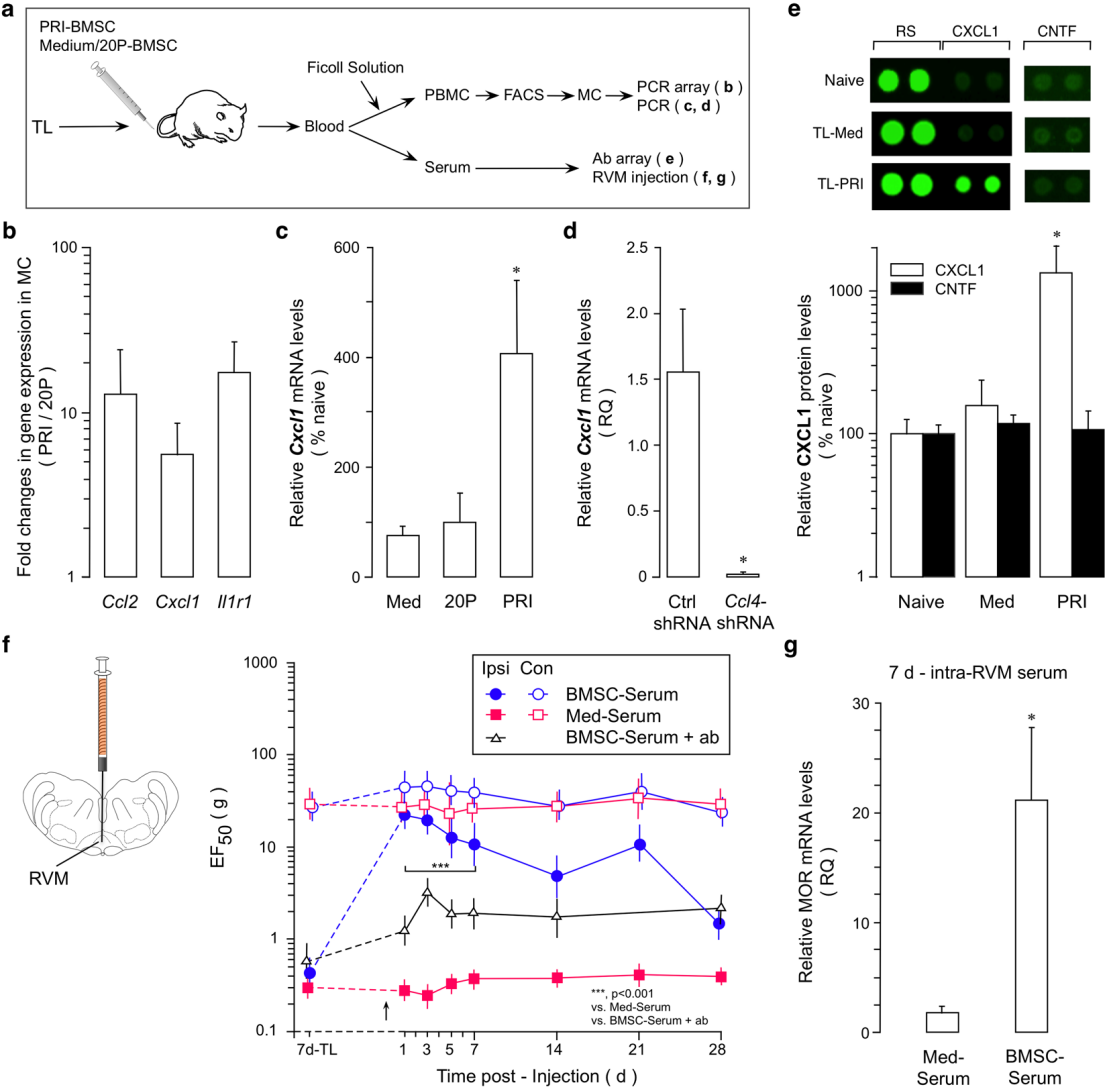
CXCL1 and antihyperalgesia. (**a**) Flow chart of the experiment. (**b**) The Rat Inflammatory Cytokines PCR Array revealed an upregulation of three genes in MC purified at 1 d after treatment with PRI BMSC in 7d-TL rats, compared to that of 20P-BMSC-treated rats. (**c**) q-PCR confirmed the upregulation of *Cxcl1* in MC. Compared to medium-(Med) and 20 P BMSC treatments, PRI-BMSC induced an increase in *Cxcl1* mRNA levels. *p < 0.05, vs. Med and 20 P, n = 3. (**d**) *Cxcl1* mRNA expression was reduced in MC of rats treated with BMSCs transduced with *Ccl4* shRNA lentivirus, compared to Ctrl shRNA treatment (n = 3, RT-qPCR). *p < 0.05, vs. ctrl shRNA. (**e**) Antibody array (ARY008, R&D) was performed on serum isolated from naïve, medium- or PRI BMSC-treated rats. There was an increased CXCL1 expression in PRI-BMSC-treated rats. CNTF (ciliary neurotrophic factor) was an example of proteins that did not show changes in expression. RS, reference spot, coated with an irrelevant biotinylated protein to show Streptavidin-HRP reaction. *p < 0.05, vs. Naïve and Med, n = 3. (**f**) Direct injection of serum (Arrow, 0.5 μl) isolated from BMSC-treated animals (BMSC-Serum) into the RVM attenuated behavioral hyperalgesia in TL rats, as compared to serum from medium-treated rats (Med-Serum). Incubation of BMSC-Serum with anti-CXCL1 antibody (ab) attenuated its antihyperalgesic effect. ***p < 0.001, vs. Med-Serum and BMSC-Serum + ab, n = 5-6/Group. (**g**). RVM MOR mRNA levels were increased in rats treated with serum isolated from BMSC-treated TL rats, compared to Med-Serum-treated rats. Sample taken at 7 d post-serum injection. *p < 0.05, n = 3. Statistics: (**b**,**c**,**e**) One-way ANOVA followed by post-hoc comparisons with Bonferroni corrections. (**d**,**g**) Unpaired, Two-tailed Student’s *t*-test. (**f**) Two-way ANOVA followed by post-hoc comparisons with Bonferroni corrections.

An intermediate role of MC in BMSC-produced antihyperalgesia raised a possibility that the pain-relieving effect of BMSCs was mediated by immune mediators from MC. We compared the gene transcription profiles of major rat inflammatory cytokines/chemokines and their receptors between MC purified from PRI- and 20P-BMSC-treated TL rats (Fig. 4a). Three genes (*Ccl2, Cxcl1 and Ilr1*) showed consistent higher levels of expression in three repeated arrays in MC isolated from PRI-BMSC-treated rats (Fig. 4b, Supplementary Fig. 7). Among the three genes exhibited higher levels, *Cxcl1* is related to regulation and release of opioids from immune cells^30, 44^. Thus, we further pursued the role of CXCL1 in BMSCs’ antihyperalgesia. The increased *Cxcl1* mRNA in PRI-BMSC-treated rats was further verified (Fig. 4c). We next confirmed that, in MC isolated from rats receiving *Ccl4* shRNA-treated BMSCs, *Cxcl1* mRNA was dramatically reduced (Fig. 4d).

CXCL1 can cross the blood-brain barrier^51^. Selective upregulation of CXCL1 in monocytes suggests that CXCL1 may be released and accesses the CNS to relay the effect of BMSCs. To test this possibility, we isolated serum from TL rats that received BMSCs or control medium treatment and observed an increase in CXCL1 in the serum derived from PRI-BMSC treated rats (Fig. 4e). Further, direct intra-RVM injection of the serum from BMSC-treated rats similarly attenuated hyperalgesia (Fig. 4f). Pre-incubation of BMSC-serum with anti-CXCL1 antibody significantly attenuated antihyperalgesia of BMSC-serum (Fig. 4g). Consistently, MOR mRNAs in the RVM were upregulated in rats treated with serum isolated from PRI-BMSC-treated rats (Fig. 4f).

### CXCR2 signaling in RVM contributes to BMSC-produced antihyperalgesia

**Figure 5:**
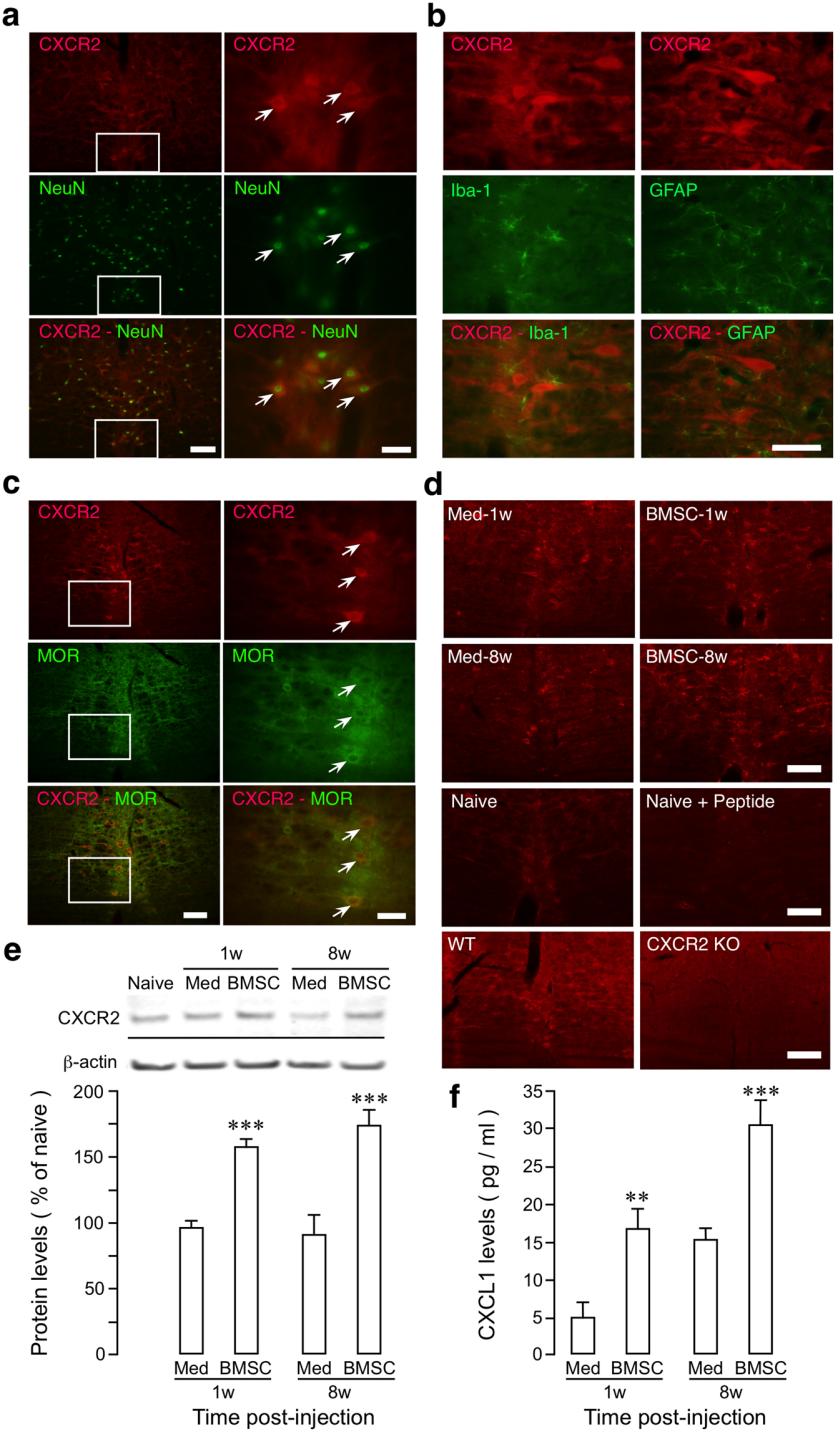
Co-localization of CXCR2 with MOR and BMSC-induced upregulation of CXCR2 in the RVM and CXCL1 in cerebrospinal fluid (CSF). (**a**) Double immunostaining showing localization of CXCR2-like immunoreactivity (red) in RVM neurons (NeuN, green). Small rectangles on the left are enlarged to the right, illustrating examples of double-labeled neurons (arrows). (**b**) Lack of colocalization of CXCR2-immunoreactivity with glial markers Iba-1 (microglia) and GFAP (astroglia). (**c**) Colocalization of CXCR2-immunoreactivity with MOR in RVM. For (**a**,**b**,**c**) Scale = 0.05 mm. (**d**) Top four images show increased CXCR2-immunoreacitivty in RVM at 1w and 8 w after primary BMSC infusion in TL rats, compared to medium-infused rats. Bottom images show CXCR2 immunostaining in naïve rats and wide type (WT) mice and loss of staining after incubation with blocking peptide for anti-CXCR2 antibody and in CXCR2 knockout mice. Scale = 0.1 mm. (**e**) TL rats received primary BMSCs or culture medium at 7d post injury and RVM tissues were collected. An example of western blot is shown on top and the relative protein levels are shown in the bottom. β-actin was a loading control. CXCR2 proteins were upregulated in RVM at 1w and 8w after injection of BMSC. ***p < 0.001, vs. Med, n = 4/group. Cropped gel images are shown. See Supplementary Figure 9 for full-length blots. (**e**) TL rats received primary BMSC or culture medium at 7d post injury and CSF was collected. Compared to medium-treated rats, CXCL1 proteins were increased in CSF at 1w and 8w after injection of BMSC. **p < 0.01; ***p < 0.001, vs. Med, n = 4/group. Statistics: One-way ANOVA followed by post-hoc comparisons with Bonferroni corrections.

CXCL1 belongs to the CXC chemokine family and signals through its receptor CXCR2. CXCR2 signaling triggers opioid release from immune cells and induces analgesia^30^. Although CXCR2 is also localized in the CNS^52, 53^, there has been no report on potential role of CXCR2 in descending pain modulation. This prompted us to explore whether CXCL1-CXCR2 signaling is involved in central mechanisms of BMSC-produced antihyperalgesia. We first examined whether CXCR2 was localized in the RVM. CXCR2-NeuN double immunostaining showed that CXCR2 was localized in RVM neurons (Fig. 5a). However, CXCR2 did not show double immunostaining with microglia marker Iba-1 (Fig. 5b, left) and astroglia marker GFAP (Fig. 5b, right). CXCR2 immunoreactivity was detected in MOR-containing neurons (Fig. 5c), indicating their colocalization in RVM. In other brain regions examined, CXCR2 immunoreactivity was found in the trigeminal ganglion, subnucleus caudalis of the spinal trigeminal complex, periaqueductal gray, but not in the periventricular nucleus of the hypothalamus (data not shown). CXCR2 immunostaining and Western blot showed that CXCR2 was upregulated in RVM at 1w and 8w after BMSC infusion (Fig. 5d,e). Further analysis of CXCL1 in cerebrospinal fluid (CSF) showed that BMSC treatment led to increased CXCL1 levels, compared to medium-treated rats (Fig. 5f), supporting increased CXCL1-CXCR2 signaling in the brain after BMSC infusion.

**Figure 6:**
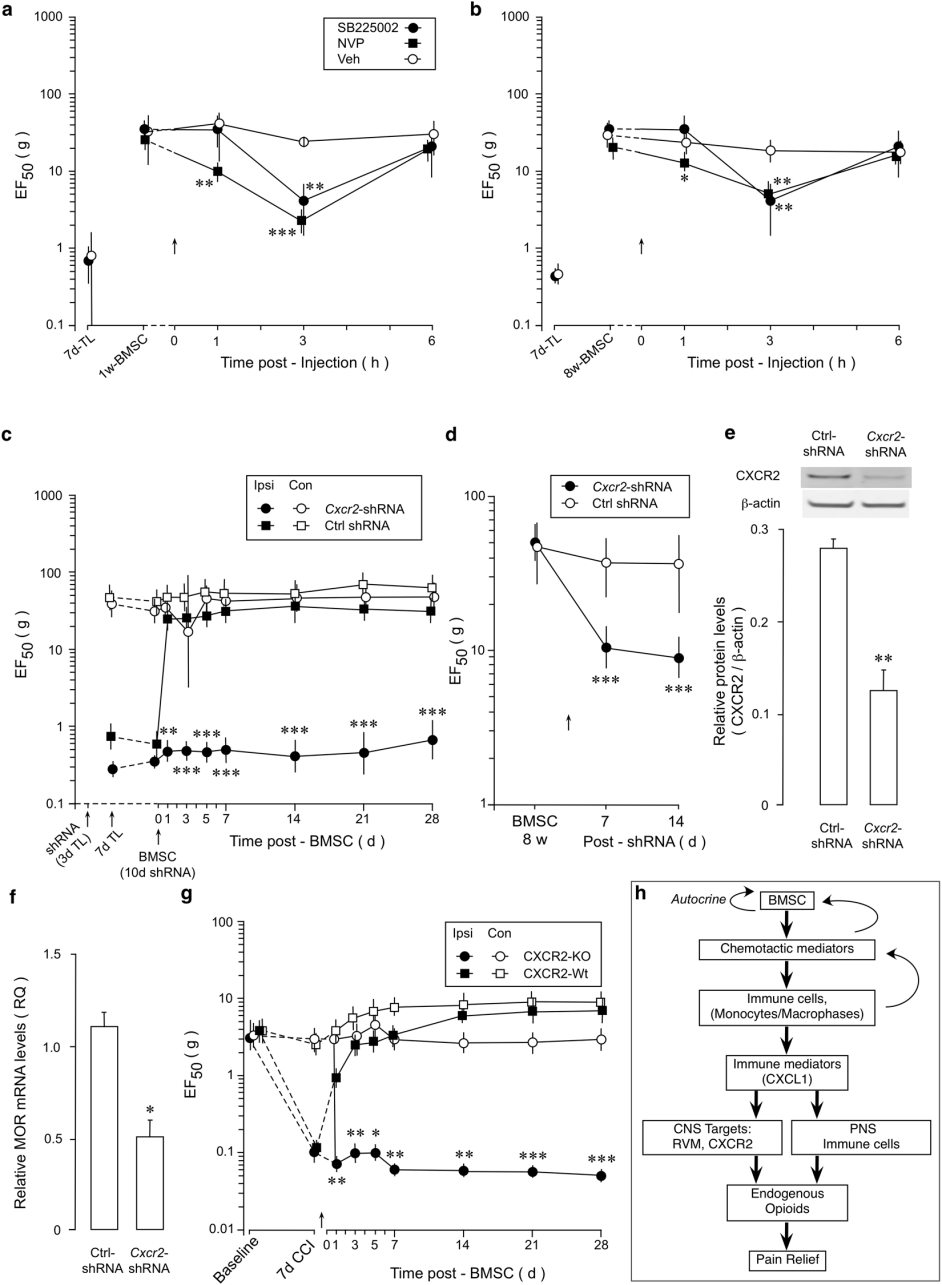
Role of RVM CXCR2 in BMSC-produced antihyperalgesia. (**a,b**) Attenuation of BMSC-induced antihyperalgesia by a CXCR2 antagonists SB225002 and NVP CXCR2 20 (NVP) injected into the RVM at 1w (**a**) and 8w (**b**) post-BMSC injection. *^-^***p < 0.05-0.001, p < 0.01; vs. Veh. (**c**) *Cxcr2* shRNA Lentivirus was microinjected into the RVM at 3d after TL and BMSCs were infused i.v. at 10d post-siRNA. Pretreatment with RNAi of *Cxcr2* attenuated BMSC-produced antihyperalgesia. (**d**) *Cxcr2* shRNA Lentivirus was microinjected into the RVM at 8w after BMSC infusion when antihyperalgesia is present. RNAi of Cxcr2 led to a reduction of EF_50_, or rekindle of hyperalgesia. ***p < 0.001, vs. Ctrl shRNA. (**e**) Western blot illustrating knock-down of CXCR2 in RVM after RNAi. Samples taken at 2w post-shRNA injection. **p < 0.01, n = 4. Cropped gel images are shown. See Supplementary Figure 9 for full-length blots. (**f**) RVM MOR mRNA levels were decreased in rats treated with *Cxcr2* shRNA, compared to Ctrl shRNA-treated rats. Sample taken at 2w post-shRNA injection. *p < 0.05, n = 4. (**g**) Mice received chronic constriction injury of the infraorbital nerve (CCI-ION) to induce hyperalgesia. The antihyperalgesic effect of BMSC was absent in the CXCR2 knockout mice. *p < 0.05, **p < 0.01, ***p < 0.001, vs. CXCR2-wt. (**a**–**d**,**g**) Error bars are 95% confidence intervals, n = 6–8/group; (**e**,**f**) Error bars are S.E.M., n = 5/group. (**h**) Summary diagram of immune interactions of BMSCs in producing pain relief. Note reciprocal interactions between BMSCs and host immune cells and autocrine signaling of BMSCs (curved arrows). Immune cells may also directly release opioid peptide and contribute to pain relief through peripheral nervous system (PNS). The role of peripheral opioid in the BMSC-produced pain relief was not studied. Statistics: (**a–d**,**g**) Two-way ANOVA followed by post-hoc comparisons with Bonferroni corrections. (**e**,**f**) Unpaired, Two-tailed Student’s *t*-test.

We then examined whether CXCR2 in the RVM plays a role in BMSC-produced antihyperalgesia. We first tested the effect of two CXCR2 antagonists SB225002 and NVP CXCR2 20 (NVP). SB225002 (100 pmol/500 nl) or NVP (200 pmol/500 nl) was injected into the RVM at 1w and 8w after infusion of BMSCs when EF_50_ on the injured side was elevated. Both antagonists produced a significant reduction of EF_50_ at the 3 h time point and NVP also reduced EF_50_ at 1 h (Fig. 6a,b), indicating attenuation of antihyperalgesia. Injection of NVP into the site dorsal to the RVM did not affect BMSC-produced antihyperalgesia (n = 4) (Supplementary Fig. 8). We then employed shRNA of *Cxcr2* in RVM. Pretreatment with *Cxcr2* shRNA Lentivirus significantly attenuated BMSC-produced antihyperalgesia (Fig. 6c). Transduction of RVM with *Cxcr2* shRNA Lentivirus at 8w post-BMSC significantly decreased EF_50_ (Fig. 6d), suggesting rekindling of hyperalgesia. Knockdown of CXCR2 by RNAi of *Cxcr2* in RVM was shown by western blot (Fig. 6e). MOR mRNA levels were decreased after CXCR2 knockdown (Fig. 6f). Finally, we tested the effect of BMSCs in CXCR2 knockout mice. The chronic constriction injury of the infraorbital nerve (CCI-ION)^54^ was produced in mice to induce persistent pain since the mouse masseter tendon was thin and vulnerable to manipulation. We have shown that BMSC attenuated pain hypersensitivity in the CCI-ION model^5^. We did not observe an antihyperalgesic effect of BMSCs in CXCR2 knockout mice while BMSC produced pain relief in the control wild type mice (Fig. 6g). These results suggest that CXCL1-CXCR2 signaling contributes to BMSC-produced long-lasting antihyperalgesia.

## Discussion

We have studied the mechanisms of BMSC-produced antihyperalgesia from a novel immune regulation and endogenous opioid perspective. Our first major finding was that, together with a pain-attenuating effect, BMSCs induced selective upregulation of MOR in the RVM, a key site for endogenous pain modulation. The finding is consistent with our hypothesis that endogenous opioids are involved in BMSCs’ antihyperalgesia^5^. This effect of BMSCs may be a global phenomenon since MOR in other pain-related regions also showed upregulation (unpublished observations). The ability of BMSCs to engage the endogenous opioid system has important implications since there has been great interest in searching for ways of activating the endogenous system for pain relief.

Importantly, we show that BMSC-produced long-lasting pain relief involved their interactions with immune cells and subsequent release of immune mediators (Fig. 6h). Our findings suggest that the maintenance of antihypergesia could be achieved by persistent chemokine signaling in the CNS. This may explain the discrepancy between the BMSCs’ lengthy pain-relieving effect and their relatively short survival in the host after systemic transplantation. In the capacity of therapeutic use of MSCs other than structural repair and replacement, more attention needs to be directed on their role as immune modulators.

Our results indicate that chemotactic interactions with host immune cells are key to BMSCs’ lasting pain relief. Compared to 20P-BMSCs that are ineffective, a number of chemokines and receptors exhibited higher levels of gene expression in pain-relieving primary BMSCs (also see ref. 29). Deletion of *Ccl4* and antagonism of CCR2 signaling in BMSCs significantly attenuated their ability to produce antihyperalgesia and MOR upregulation. Both CCL4-CCR5 and CCL2-CCR2 signaling pathways are involved in monocyte chemotaxis^32, 40^. Thus, chemotactic interactions in the circulation may occur immediately after BMSC infusion and subsequent down-stream effect in host immune cells are critical of BMSCs’ antihyperalgesia. It should be cautioned that potential roles of additional chemokines cannot be ruled out since chemokines function in a redundant and overlapping fashion^55^ and *in vivo* interactions of BMSCs should involve a cohort of chemoattractants and their receptors.

Thus, BMSC-derived mediators apparently require collaboration of host immune system in producing antihyperaglesia. Our monocytes/macrophages depletion experiments suggest that monocytes/macrophages are targeted by BMSCs to relay their biological effect. After systemic infusion, BMSCs travel to the lungs and show close relationship to macrophages^22^, suggesting a site of interaction between BMSCs and monocytes/macrophages. Monocytes from BMSC-treated animals exhibited selective upregulation of CXCL1 chemokine that is related to opioid release from immune cells^30, 44^. Intriguingly, CXCL1 also showed an increased level in the serum and CSF derived from BMSC-treated TL rats and direct injection of this serum upregulated MOR in RVM and mimicked BMSC’s antihyperalgesia. This raises a possibility that BMSC-induced immune mediators may access the brain to facilitate antihyperalgesia. Indeed, CXCL1 readily penetrates the blood brain barrier to reach the brain parenchyma in a matter of minutes^51^.

CXCL1 initiates its biological effect through the receptor CXCR2 that is also localized in the CNS^52, 53^. Antagonism of CXCR2 in RVM counteracted BMSC-produced MOR upregulation and antihyperalgesia, suggesting a critical role of CXCL1-CXCR2 signaling in BMSC-produced pain relief. Relevant to these observations, CXCL1 is neuroprotective during autoimmune demyelination^56^, facilitates opioid analgesia^43^, and potentially antinociceptive against neuropathic pain^57^. We noticed that studies have shown hyperalgesic action of CXCL1-CXCR2 signaling^52, 58, 59^. These phenomena should be understood with a view that the same immune mediators involved in pain sensitization may also be neuroprotective and promote pain relief under certain conditions^60, 61^. Similar dual roles of the same mediator in pain and analgesia have also been observed. Glucocorticoids compounds are anti-inflammatory through activation of their receptors, but activation of glucocorticoid receptors may also be proinflammatory and pronociceptive after injury^62^. Endothelin-B receptors are pro-algesic along with the ability to activate analgesic pathway^63^. While the anti-inflammatory cytokines and receptors *Tgfβ*, *IL10 and IL10ra* showed higher expression in primary BMSCs, consistent with BMSCs’ pain relieving effect, genes of proinflammatory cytokines *Tnf*, *IL1β* and *IL18* and complement components *C3* and *C5* also exhibited higher levels of expression in primary BMSCs. In monocytes isolated from BMSC-treated rats, proinflammatory CCL2 and IL1R showed higher levels of expression. The presence of these inflammatory factors may contribute to the effect of BMSCs by promoting chemotactive interactions of BMSCs with immune cells^21^. Further, TNF may stimulate MOR gene expression in neurons^42^ and CXCR2 related ligands CXCL1/2/5 may induce opioid release from peripheral immune cells^43^.

Although our results suggest positive interactions between CXCR2 and MOR, this is rather unexpected. The interaction between chemokine receptors and opioid receptors often leads to heterologous desensitization involving formation of heterodimers^64^, although CXCR2 has been shown not involved in MOR desensitization^65^. The nature of potential interactions between CXCL1-CXCR2 signaling and MOR in the event of BMSC intervention under injurious conditions requires further study.

## Methods

### Animals

Sprague-Dawley rats (200-250 g) were purchased from Harlan, Indianapolis, IN. GFP transgenic S-D rats [SD-Tg(GFP)Bal] were purchased from University of Missouri Rat Resource and Research Center. The breeder pair CXCR2 knockout [C.129S2(B6)-Cxcr2^tm1Mwm^/J] and wild type control (BALB/c) mice were from The Jackson laboratory and bred in the Animal Facility of the University of Maryland School of Dentistry. Genotyping was performed by Geno Typing Center of America (Bangor, ME). All behavioral tests were conducted under blind conditions. The rat brain atlas^66^ was referred to for identifying and approaching brain structures. All experiments were carried out in accordance with the National Institute of Health Guide for the Care and Use of Laboratory Animals (NIH Publications No. 80-23) and approved by the University of Maryland, Baltimore, Institutional Animal Care and Use Committee, School of Dentistry and School of Medicine.

### Data analysis

Power analyses were performed based on our previous data to determine the necessary number of animals needed to provide an 80% chance to achieve statistical significance (alpha = 0.05) and minimal number of animals were used in some experiments when statistical significance was achieved. Data are presented as mean (95% confidence interval) for EF_50_s and mean ± S.E.M. for other data. Statistical comparisons were made by ANOVA followed by post-hoc comparisons with Bonferroni corrections. For unbalanced sample size, multiple regressions were performed and Type III sum-of-squares was used to calculate P values (GraphPad Prism). For animals that were subject to repeated testing, ANOVA with repeated measures was used. P < 0.05 was considered significant for all cases.

Additional Methods are available in the online version of the paper.

### Data availability

The datasets generated during and/or analyzed during the current study are available from the corresponding author on reasonable request.

## Electronic supplementary material

Supplementary materials
