# Supplementary material for: In vivo immune interactions of multipotent stromal cells underlie their long-lasting pain-relieving effect
Source: Sci Rep. 2017 Aug 31;7:10107. doi: 10.1038/s41598-017-10251-y (PMC5579160; doi:10.1038/s41598-017-10251-y)

***In vivo* immune interactions of multipotent stromal cells underlie their long-lasting pain-relieving effect**

Wei Guo[1+], Satoshi Imai[1,2+], Jia-Le Yang[1], Shiping Zou[1], Mineo Watanabe[1,3], Yu-Xia Chu[1,4], Zaid Mohammad[1], Huakun Xu[5], Kamal D. Moudgil[6], Feng Wei[1], Ronald Dubner[1], Ke Ren[1*]

[1]Department of Neural and Pain Sciences, School of Dentistry, & Program in Neuroscience, University of Maryland, Baltimore, MD 21201, USA

[2]Present Affiliation: Department of Clinical Pharmacology and Therapeutics, Kyoto University Hospital, 54 Shogoin Kawahara-cho, Sakyo-ku, Kyoto 606-8507, Japan

[3]Present Affiliation: Department of Orthodontics and Craniofacial Developmental Biology, Hiroshima University, Graduate School of Biomedical Sciences, 1-2-3 Kasumi, Minami-ku, Hiroshima, 734-8553, Japan

[4]Present Affiliation: Department of Integrative Medicine and Neurobiology, School of Basic Medical Sciences, Fudan University, Shanghai, China

[5]Division of Biomaterials and Tissue Engineering, School of Dentistry, University of Maryland, Baltimore, MD 21201, USA

[6]Department of Microbiology & Immunology, University of Maryland, Baltimore, MD 21201, USA

[+], These authors contributed equally to this work

*Correspond to:

K. Ren

Dept of NPS, 650 W. Baltimore St., Dental-8 South, Baltimore, MD 21201, USA

Tel: 01 410 706 3250, Fax: 01 410 706 0865, E-mail: kren@umaryland.edu

Supplemental Information

METHODS

Animal models

All surgical procedures were performed under pentobarbital sodium (50 mg/kg i.p.) anesthesia. For the rat tendon ligation (TL) model, TL was achieved via an intraoral approach as described[5]. On the left intraoral site, a five-mm long incision was made posterior-anteriorly lateral to the gingivobuccal margin in the buccal mucosa, beginning immediately next to the first molar. The tendon of the anterior superficial part of the rat masseter muscle was gently freed and tied with two chromic gut (4.0) ligatures, 2-mm apart. The chronic constriction injury of the infraorbital nerve (CCI-ION) mouse model was produced according to Wei et al.[54] A 5-7 mm long incision was made along the gingivobuccal margin in the buccal mucosa. The ION is freed from surrounding connective tissues. At 3-4 mm from the nerve where its branches emerge from the infraorbital fissure, the ION was loosely tied with two chromic gut (4.0) ligatures, 2-mm apart.

Behavioral testing

All behavioral tests were conducted under blind conditions. Mechanical sensitivity of the orofacial region was assessed as described elsewhere[5,45]. A series of calibrated von Frey filaments were applied to the skin above the injured tendon or the corresponding contralateral side. An active withdrawal of the head from the probing filament was defined as a response. Each von Frey filament was applied 5 times at intervals of 5-10 seconds. The response frequencies [(number of responses/number of stimuli) X100%] to a range of von Frey filament forces were determined and a stimulus-response frequency (S-R) curve plotted. After a non-linear regression analysis, an EF_{50} value, defined as the effective von Frey filament force (g) that produces a 50% response frequency, was derived from the S-R curve (Prism, GraphPad)[5]. A leftward shift of the S-R curve, resulting in a reduction of EF_{50}, occurred after TL. This shift of the curve suggests the development of mechanical hypersensitivity, or presence of mechanical

hyperalgesia and allodynia since there was an increase in response to suprathreshold stimuli and a decreased response threshold for nocifensive behavior.

BMSC procedures

BMSCs were obtained from donor rats as described[5]. The rats were sacrificed with CO_2, the both ends of the tibiae, femurs and humeri were cut off by scissors. A syringe fitted with 18-gauge needle was inserted into the shaft of the bone and bone marrow was flushed out with culture medium (alpha–modified Eagle medium, Gibco, Carlsbad, CA, USA; 10% fetal bovine serum, Hyclone, Logan, UT, USA). The bone marrow was then mechanically dissociated and the suspension passed through a 100-μm cell strainer to remove debris. The cells were incubated at 37°C in 5% CO_2 in tissue-culture flasks (100 X 200 mm) (Sarstedt, Nümbrecht, Germany), and non-adherent cells removed by replacing the medium. When the cultures reached 80% confluence, the cells were washed with PBS and harvested. The cell numbers were calculated by the Hemacytometer. For intravenous administration, 1.5 X 10^6 cells (1.5 M) in 0.2 ml PBS were slowly injected into one tail vein of the anesthetized animal over a 2-minute period using a 22-gauge needle. The property of expanded cells was assessed by flow cytometry with conventional markers[5].

Cryopreserved human primary BMSC (hBMSC) were purchased (KT-002, RoosterBio, Inc., Frederick, MD, USA). hBMSC vial from liquid nitrogen was immediately thawed in 37°C water bath. Cells were removed from water bath once only a small bit of ice was remaining. Cells were aseptically transferred into 50-ml centrifuge tube, and 4 ml of culture media were slowly added. Cells were centrifuged 200Xg for 10 min and the supernatant was carefully removed without disturbing the pellet. The cells were then resuspended in 45 ml of culture media, mixed well and seeded into three T75 vessels. After reaching 90% confluence the cells were passed into 6 10-cm culture plates. The cells were collected after reaching more than 90% confluence, and 1.5 M cells infused to the animals.

Drugs and antibodies

The opioid receptor antagonist 6-β-naltrexol, CCR2 receptor antagonist RS-102895 hydrochloride {1'-[2-[4-(Trifluoromethyl)phenyl]ethyl]-spiro[4H-3,1-benzoxazine-4,4'-peperidin]-2(1H)-one}, and CXCR2 antagonist SB225002 [N-(2-Bromophenyl)-N'-(2-hydroxy-4-nitrophenyl)urea, were from Sigma-Aldrich (St. Louis, MO). The CXCR4 antagonist AMD3100 (plerixafor) {1,1'-[1,4-Phenylenebis-(methylene)]-bis-(1,4,8,11-tetraazacyclotetradecane) octahydrochloride} and CXCR2 antagonist NVPCXCR2 20 {4-Cyclopropyl-2-[[(2,3-difluorophenyl)methyl]thio]-1,6-dihydro-6-oxo5-pyrimidinecarbonitrile} were from Tocris Bioscience (Bristol, UK).

Antibodies used were rabbit anti-MOR (1:1,000 for Western blot; 1:1,500 for immunohistochemistry) from Immunostar (Hudson, WI); mouse anti-beta-actin (1:5,000 for western blot) from Sigma-Aldrich; mouse FITC-conjugated anti-CD90.1 (clone OX-7) (1:100 for immunohistochemistry) from BD Pharmingen; mouse anti-CD11b (clone OX-42, 1:500 for Immunocytochemistry) from AbD serotec (Raleigh, NC); mouse anti-CD11b (clone OX-42, 1:100 for flow cytometry) from eBioscience; mouse anti-CD68 (clone ED1, 1:500 for immunocytochemistry) from Acris Antibodies Gmbh (Herford, Germany); rabbit anti-CXCR2 (1:400 for immunocytochemistry) and rabbit anti-CCR2 (1:1,000 for western blot) from abcam (Cambridge, UK); rabbit anti-CXCR2 (1:1,000 for western blot) from Boster Biological Tech. (Pleasanton, CA); sheep anti-CCL4 (1:1000 for western blot) from R&D Systems (Minneapolis, MN); mouse anti-NeuN (clone A60, 1:10,000 for immunohistochemistry) and mouse anti-GFAP (clone GA5, 1:1,000 for immunohistochemistry) from EMD Millipore, (Darmstadt, Germany); and rabbit anti-Iba-1 (1:2,000 for immunohistochemistry) from Wako Chemicals (Richmond, VA). Synthetic blocking peptide (Amino acids 18-39: EDLSNYSYSSTLPPFLLDAAPC) for anti-CXCR2 antibody was from abcam.

RT-qPCR.

Total RNAs were extracted using mirVana™ miRNA Isolation Kit (Applied Biosystems). The purified total RNA was quantified at 260 nm with NanoDrop 2000 (Thermo Fisher Scientific). To prepare first strand cDNA, 1.0 µg of RNA was incubated in 100 µl of buffer containing 10 mM dithiothreitol, 2.5 mM MgCl2, dNTP mixture, 200 units of reverse transcriptase II and 0.1 mM oligo (dT)12−18 (Life Technologies). Each gene prepared by the above procedure was amplified in 20 µl of a PCR solution containing 10 µl of Power SYBR® Green PCR Master Mix (Applied Biosystems) with synthesized primers. PCR with the StepOnePlus™ System (Applied Biosystems) was performed with the following cycling conditions: 95˚C for 10 min and 60˚C for 1 min, followed by cycled 40 cycles of 95˚C for 15 sec and 60˚C for 1 min. Fluorescence detection was conducted after each extension step. GAPDH was used as a normalization control, and relative mRNA levels were calculated by a comparative Ct or ΔΔCt method using StepOnePlus™ software. For semi-quantitative comparison, samples were either further normalized to naïve control or a control sample was used as a calibrator to obtain RQ (relative quantification) values.

The PCR primers were *Oprm1 (MOR)*, 5'-TCCCACCCAACCTGGTACTG-3' and 5'-TAGCATGCGAACGCTCTTGA-3'; *Oprd1* (DOR), 5'-AACGTGCTCGTCATGTTTGG-3' and 5'-CAGGTACTTGGCGCTCTGGAA-3'; *Oprk1* (KOR), 5'-GCATTTGGCTACTGGCATCA-3' and 5'-GGAAACTGCAAGGAGCATTCA-3'; *gapdh*, 5’-GCAAGAGAGAGGCCCTCAG-3’ and 5’-TGTGAGGGAGATGCTCAGTG-3’; *Actb*, 5'-ACTATCGGCAATGAGCGGTTCC-3' and 5'-CTGTGTTGGCATAGAGGTCTTTACG-3'; *Pf4 (Cxcl4)*, 5'-GCTGTGTGTGTGTGAAGACCAG-3' and 5'-CATTCTTCAGCGTGGCTATGAG-3'; *Ccl4*, 5'-CCTTCTCTCTCCTCCTGCTTGT-3' and 5'-GAATCTTCCGGGAGGTGTAAGA-3'; *Ccl12*, 5'-AGATCCACATTCGGAGGCTAAA-3' and 5'-GGGTCAGCACAGAGCTCCTTAT-3'; *Tnf*, 5'-AAATGGGCTCCCTCTCATCAGTTC-3' and 5'-TCCGCTTGGTGGTTTGCTACGAC-3'; *Ccr5*, 5'-TGACACACTGCTGCCTCAACCCT-3' and 5'-TCCTGTTCTCCTGTGGACCGGGTA-3'; *Ccr2*, 5'-TATGCTGCAAATGAGTGGGTCT-3' and 5'-

AAAGCAAAGACAGCATGGACAA-3'; *Ccl2*, 5'-CAGATCTCTCTTCCTCCACCACTAT-3' and 5'-CAGGCAGCAACTGTGAACAAC-3'; *Cxcl12*, 5'-TCTGCATCAGTGACGGTAAGC-3' and 5'-TGAAGGGCACAGTTTGGAGT-3'; *Ccl5*, 5'-ACTCCCTGCTGCTTTGCCTACC-3' and 5'-TTGGCGGTTCCTTCGAGTGAC-3'; *Tgfb1*, 5'-TGAGTGGCTGTCTTTTGACG-3' and 5'-TGGGACTGATCCCATTGATT-3'; *Irf4*, 5'-AGCTGGATATCTCCGACCCATA-3' and 5'-CATAAGGTGCTGTCATGGGGTA-3'; *Cd206*, 5'-CGACCCTTCTGTGCCTATCTCT-3' and 5'-CCATGGCGACTTCAATTTCATA-3'; *Cd163*, 5'-CAATGAAGCCCACAGAGTGAAC-3' and 5'-AACTCCTTTTGGACACTGCACA-3'; *Il-10*, 5'-CACTGCTATGTTGCCTGCTCTTAC-3' and 5'-GGGTCTGGCTGACTGGGAAG-3'; *Irf5*, 5'-CAGCCCCCTACAGAACAACTCT-3' and 5'-CCAAAGAGTTCCACTTGCTCCT-3'; *Cd86*, 5'-GCCCGAGTGAGCTCGTAGTATT-3' and 5'-AATCATACAAGCCCGTGTCCTT-3'; *Il-6*, 5'-CAAGAGACTTCCAGCCAGTTGC-3' and 5'-TGTTGTGGGTGGTATCCTCTGTG-3'; *Il-1b*, 5'-GAAAGACGGCACACCCACC-3' and 5'-AAACCGCTTTTCCATCTTCTTCT-3'; *Cxcl10*, 5'-GGGCCATAGGAAAACTTGAAATC-3' and 5'-CATTGTGGCAATGATCTCAACA-3'; *Ccl2*, 5'-CAGATCTCTCTTCCTCCACCACTAT-3' and 5'-CAGGCAGCAACTGTGAACAAC-3'; *Cxcl1*, 5'-GTGGCAGGGATTCACTTCAAG-3' and 5'-CCATCGGTGCAATCTATCTTC-3'; and *Ilr1*, 5'-TAGATGACAGCAAGAGGGA-3' and 5'-ACTTCCAGTAGACAAGGTC-3'.

Western blot

Rats were anesthetized with isoflurane (3%) and quickly decapitated. The brainstem tissue block that included rostral ventromedial medulla (RVM) was harvested by taking punches with a 15-gauge needle. Tissues were homogenized in solubilization buffer (50 mM Tris.HCl, pH8.0; 150 mM NaCl, 1 mM EDTA, 1% NP40, 0.5% deoxycholic acid, 0.1% SDS, 1 mM Na_3VO_4, 1 U/ml aprotinin, 20 μg/ml leupeptin, 20 μg/ml pepstatin A). The homogenate was centrifuged at 20,200 X g for 10 min at 4°C. The supernatant was removed. The protein concentration was determined using a detergent-compatible protein assay with a bovine serum albumin standard.

For detecting the immunoreactivity with near-infrared fluorescence using the Odyssey Infrared Imaging System (Odyssey®CLx, LI-COR, Lincoln, NE), 50 μg protein samples were denatured by boiling for 5 min and loaded onto 4-20% Bis-Tris gels (Invitrogene). After electrophoresis, proteins were transferred to nitrocellulose membranes. The membranes were blocked for 1 h with Odyssey Blocking Buffer and then incubated with primary antibodies diluted in Odyssey Blocking Buffer at 4°C overnight, followed by washing with PBS containing 0.1% Tween 20 (PBST) three times. The membranes were then incubated for 1 h with IRDye800CW-conjugated goat anti-rabbit IgG and IRDye680-conjugated goat anti-mouse IgG secondary antibodies (LI-COR) diluted in Odyssey Blocking Buffer. The blots were further washed three times with PBST and rinsed with PBS. Proteins were visualized by scanning the membrane with 700- and 800-nm channels. The loading and blotting of the amount of protein was verified by reprobing the membrane with anti-β-actin and with Coomassie blue staining.

Immunocytochemistry

Rat blood monocytes (2 x 10^6 cells/well) or rat BMSC (5 x 10^5 cells/well) were cultured on Nunc™ Lab-Tek™ 8 wells Chamber Slide System (Thermo Fisher Scientific). At 1 d after the seeding of cells, the culture medium was removed, and the cells were washed 3 times with PBS and fixed with 4% paraformaldehyde in PBS for 20 min at RT. Cells were washed 3 times with PBS, permeabilized with 0.1% Triton X-100 in PBS for 20 min at RT, and then blocked with 2% BSA in PBS for 30 min at RT. After washing 3 times with PBS, monocytes or BMSC were incubated with relevant primary antibodies overnight at 4°C. All primary antibodies were diluted with 2% BSA in PBS. After three times washing with PBS, cells were incubated with appropriate secondary antibodies IgG-Cy2 and/or IgG-Cy3 (1:500) for 2 h at RT. After washing, cultures were mounted using ProLong® Gold Antifade Reagent with DAPI (Cell Signaling Technology).

Immunohistochemistry

Rats were deeply anesthetized with pentobarbital sodium (100 mg/kg, i.p.) and perfused transcardially with 4% paraformaldehyde in 0.1 M phosphate buffer at pH 7.4. The same block of caudal brainstem tissues as that for western blot was removed, post-fixed, and transferred to 25-30% sucrose (w/v) for cryoprotection. Free-floating tissue sections (30-µm thick) were incubated with relevant antibodies with 1-3% relevant normal sera and single or double-labeling immunofluorescence was performed. Double-labeling immunofluorescence was performed with Cy2 and Cy3 (1:500, Jackson ImmunoResearch) or Alexa Fluor 488 (1:500, Invitrogen Molecular Probes) and Alexa Fluor 568 (1:500) after incubation with respective primary antibodies. Biotin-SP donkey anti-rabbit IgG (1:600, Jackson ImmunoReserch) and streptavidin Alexa Fluor 568 conjugate (1:600, Invitrogen Molecular Probes) were also used in some experiments. Control sections were processed with the same method except that the primary antibodies were omitted or adsorbed by respective antigens. The tyramide signal amplification (TSA) was used for double immunofluorescence of CXCR2 with MOR, NeuN, Iba-1 and GFAP.

ELISA

Rat CXCL1 Quantikine ELISA (R&D Systems) was performed on cerebrospinal fluid collected from medium- or PRI BMSC-treated TL rats according the manufacturer's protocol. Cerebrospinal fluid (0.15 ml/rat) was collected through atlantooccipital membrane under isoflurane anesthesia.

RT^2 PCR Array

Total RNAs from primary and 20P BMSCs as well as peripheral blood monocytes derived from BMSC or culture medium-treated rats were characterized in triplicates using The Rat Inflammatory Cytokines & Receptors RT^2 Profiler^{TM} PCR Array (PARN-011A, Qiagen) following

the manufacturer's protocol. Total RNAs were extracted using mirVana™ miRNA Isolation Kit (Applied Biosystems). Purified 1 μg of total RNA was used to prepare first-strand cDNA. The array was probed according to the manufacture's instruction in the StepOnePlus™ System (Applied Biosystems). Gene expression data were analyzed with the web-based RT^2 Profiler PCR Array Data Analysis software that performed ΔΔCt based fold-change calculations from the uploaded threshold cycle data (http://pcrdataanalysis.sabiosciences.com/pcr/arrayanalysis.php?target=upload).

The following genes were included in the RT^2 Profiler™ PCR Array:

Accession No.	Symbol	Description
NM_001109883	*Abcf1*	ATP-binding cassette, subfamily F (GCN20), member 1
NM_001107084	*Bcl6*	B-cell CLL/lymphoma 6
NM_053303	*Cxcr5*	Chemokine (C-X-C motif) receptor 5
NM_016994	*C3*	Complement component 3
XM_345342	*C5*	Complement component 5
NM_012762	*Casp1*	Caspase 1
NM_019205	*Ccl11*	Chemokine (C-C motif) ligand 11
NM_001105822	*Ccl12*	Chemokine (C-C motif) ligand 12
NM_057151	*Ccl17*	Chemokine (C-C motif) ligand 17
NM_001108661	*Ccl19*	Chemokine (C-C motif) ligand 19
NM_031530	*Ccl2*	Chemokine (C-C motif) ligand 2
NM_019233	*Ccl20*	Chemokine (C-C motif) ligand 20
NM_001008513	*Ccl21*	Chemokine (C-C motif) ligand 21
NM_057203	*Ccl22*	Chemokine (C-C motif) ligand 22
NM_001013045	*Ccl24*	Chemokine (C-C motif) ligand 24
NM_001037203	*Ccl25*	Chemokine (C-C motif) ligand 25
NM_013025	*Ccl3*	Chemokine (C-C motif) ligand 3
NM_053858	*Ccl4*	Chemokine (C-C motif) ligand 4
NM_031116	*Ccl5*	Chemokine (C-C motif) ligand 5
NM_001004202	*Ccl6*	Chemokine (C-C motif) ligand 6
NM_001007612	*Ccl7*	Chemokine (C-C motif) ligand 7
NM_001012357	*Ccl9*	Chemokine (C-C motif) ligand 9
NM_020542	*Ccr1*	Chemokine (C-C motif) receptor 1
NM_021866	*Ccr2*	Chemokine (C-C motif) receptor 2
NM_053958	*Ccr3*	Chemokine (C-C motif) receptor 3
NM_133532	*Ccr4*	Chemokine (C-C motif) receptor 4
NM_053960	*Ccr5*	Chemokine (C-C motif) receptor 5
NM_001013145	*Ccr6*	Chemokine (C-C motif) receptor 6
NM_199489	*Ccr7*	Chemokine (C-C motif) receptor 7
XM_236704	*Ccr8*	Chemokine (C-C motif) receptor 8
NM_172329	*Ccr9*	Chemokine (C-C motif) receptor 9
NM_017096	*Crp*	C-reactive protein, pentraxin-related
NM_134455	*Cx3cl1*	Chemokine (C-X3-C motif) ligand 1
NM_133534	*Cx3cr1*	Chemokine (C-X3-C motif) receptor 1

NM_030845	*Cxcl1*	Chemokine (C-X-C motif) ligand 1
NM_139089	*Cxcl10*	Chemokine (C-X-C motif) ligand 10
NM_182952	*Cxcl11*	Chemokine (C-X-C motif) ligand 11
NM_022177	*Cxcl12*	Chemokine (C-X-C motif) ligand 12 (stromal cell-derived factor 1)
NM_053647	*Cxcl2*	Chemokine (C-X-C motif) ligand 2
NM_022214	*Cxcl5*	Chemokine (C-X-C motif) ligand 5
NM_145672	*Cxcl9*	Chemokine (C-X-C motif) ligand 9
NM_053415	*Cxcr3*	Chemokine (C-X-C motif) receptor 3
NM_001108836	*Ccr10*	Chemokine (C-C motif) receptor 10
NM_138880	*Ifng*	Interferon gamma
NM_012854	*Il10*	Interleukin 10
NM_057193	*Il10ra*	Interleukin 10 receptor, alpha
NM_133519	*Il11*	Interleukin 11
NM_053828	*Il13*	Interleukin 13
NM_145789	*Il13ra1*	Interleukin 13 receptor, alpha 1
NM_013129	*Il15*	Interleukin 15
NM_001105749	*Il16*	Interleukin 16
NM_053789	*Il17b*	Interleukin 17B
NM_019165	*Il18*	Interleukin 18
NM_017019	*Il1a*	Interleukin 1 alpha
NM_031512	*Il1b*	Interleukin 1 beta
NM_001107814	*Il1f5*	Interleukin 1 family, member 5 (delta)
NM_001106554	*Il1f6*	Interleukin 1 family, member 6
NM_013123	*Il1r1*	Interleukin 1 receptor, type I
NM_053953	*Il1r2*	Interleukin 1 receptor, type II
NM_013195	*Il2rb*	Interleukin 2 receptor, beta
NM_080889	*Il2rg*	Interleukin 2 receptor, gamma
NM_031513	*Il3*	Interleukin 3
NM_201270	*Il4*	Interleukin 4
NM_021834	*Il5*	Interleukin 5
NM_053645	*Il5ra*	Interleukin 5 receptor, alpha
NM_017020	*Il6r*	Interleukin 6 receptor
NM_001008725	*Il6st*	Interleukin 6 signal transducer
NM_019310	*Il8ra*	Interleukin 8 receptor, alpha
NM_017183	*Cxcr2*	Chemokine (C-X-C motif) receptor 2
NM_012711	*Itgam*	Integrin, alpha M
NM_001037780	*Itgb2*	Integrin, beta 2
NM_080769	*Lta*	Lymphotoxin alpha (TNF superfamily, member 1)
NM_212507	*Ltb*	Lymphotoxin beta (TNF superfamily, member 3)
NM_031051	*Mif*	Macrophage migration inhibitory factor
NM_001007729	*Pf4*	Platelet factor 4, Chemokine (C-X-C motif) ligand 4
NM_053757	*Aimp1*	Aminoacyl tRNA synthetase complex-interacting multifunctional protein 1
NM_012881	*Spp1*	Secreted phosphoprotein 1
NM_021578	*Tgfb1*	Transforming growth factor, beta 1
NM_012675	*Tnf*	Tumor necrosis factor
NM_013091	*Tnfrsf1a*	Tumor necrosis factor receptor superfamily, member 1a
NM_130426	*Tnfrsf1b*	Tumor necrosis factor receptor superfamily, member 1b
NM_053353	*Cd40lg*	CD40 ligand
NM_001109668	*Tollip*	Toll interacting protein
NM_001106871	*Xcr1*	Chemokine (C motif) receptor 1

Proteome Profiler™ Array

Rat cytokine antibody array (ARY008, R&D Systems) was performed on serum isolated from naïve, medium- or PRI BMSC-treated TL rats according the manufacturer's protocol. The serum (0.2 ml) was added to the array membranes coated with 29 specific cytokine antibodies. The membranes were incubated at 4°C overnight. Array images were collected and analyzed using the LI-COR Odyssey Infrared Imaging System. The relative protein levels were obtained by subtracting the background staining and normalizing to the positive controls on the same membrane. We paid special attention to the level of CXCL1 and the results of other cytokines are not shown.

RNAi

Ccl4 shRNA (Accession Number NM_053858.1 CDS, target sequence: 90: TCCCACTTCCTGCTGCTTCTCTTACACCT) was transduced into cultured BMSC [*Ccl4* RNAi lentivirus (piLenti-siRNA-GFP, abm® Richmond, BC, Canada]. BMSCs were plated onto 10-cm plate before transduction. When they reached 80% confluence 5 ml medium (without serum) was added with 4 µl polybrene (8 µg/ml) and 50 µl of ViralPlus Transduction Enhancer G698 (abm). Eighty-µl *Ccl4* shRNA lentivirus or control shRNA lentivirus was then added to the plate. Cells were incubated at 37°C with 5% CO_2 and collected at 72 h following transduction. *Cxcr2* shRNA (Accession Number NM_017183 CDS, target sequences: (118: TCAGCGAACCTAGATATCAACAGGTATGC, 306: CTGGGCTGCATCTAAAGTAAATGGATGGA, 829: AGAACCAAGCTGATCAAGGAGACCTGTGA, 970: CTTCTCAAGATCATGGCTAATTATGGCCT) Lentivirus (abm) was microinjected into the RVM in 500 nl.

Monocyte/macrophage procedures

Clodronate (Cl_2MDP, dichloromethylene diphosphnate)-filled liposomes (Lipo-Clo) were prepared as described[45] or purchased from clodronateliposomes.com (Amsterdam, Netherlands). Liposome-encapsulated Cl_2MDP (2 ml) was injected i.v. once per day for three days. The effects of the Lipo-Clo treatment on macrophages were verified with flow cytometry (peritoneal macrophages) and immunohistochemistry (spleen) with CD11b and CD68, respectively. For isolation of macrophages, 3% thioglycolate was injected i.p. for three days and 20 ml of minimum essential medium (MEM) alpha medium (Gibco) with 10% fetal calf serum was injected i.p. at 30 minutes before isolation. Peritoneal cells were collected with a 3-ml syringe, plated on culture dishes, and kept at 37°C overnight.

Monocytes isolation

Heparinized rat blood was overlaid on Ficoll solution (Ficoll-Paque Plus, GE Life Sciences) and centrifuged at 500 x g for 30 min. The monocyte/lymphocyte/basophil pellicle was removed and further separated and purified by FACS with anti-CD11b antibodies. Flow cytometry was performed at the University of Maryland Greenbaum Cancer Center Shared Flow Cytometry Facility. Cell fluorescence was evaluated by flow cytometry in an FACScan (Beckton Dickinson, San Jose, CA) analytical flowcytometer and the data were analyzed using FlowJo 8.8.6 (TreeStar, Inc., Ashland, OR). The specific staining was measured from the cross point of the isotype control with a specific antibody graph.

Brainstem microinjections[5]

Rats were anesthetized with 2–3% isoflurane in a gas mixture of 30% O_2 balanced with 70% nitrogen and placed in a Kopf stereotaxic instrument (Kopf Instruments, Tujunga, CA). A midline incision was made after infiltration of lidocaine (2%) into the skin. A midline opening was made in the skull with a dental drill for inserting an injection needle into the target site. The coordinates for the rostral ventromedial medulla (RVM) were: 10.5 mm caudal to the bregma,

midline and 9.0 mm ventral to the surface of the cerebellum[66]. Microinjections were performed by delivering drug solutions slowly over a 10-min period using a 500 nl Hamilton syringe with a 32-gauge needle. The injection needle was left in place for at least 15 min before being slowly withdrawn. The wound was closed and animals were returned to their cages after recovering from anesthesia. For histology verification of the injection site, 30-μM coronal brainstem sections were stained with Neurotrace™ 500/525 Green fluorescent Nissal Stain (Invitrogen) (1:500 for 20 min).

Supplemental Figure Legends

Supplementary Fig. 1. a,b. Effect of a neutral opioid receptor antagonist 6-β-naltrexol on BMSC-produced attenuation of mechanical hypersensitivity. **a.** BMSC attenuated hyperalgesia in rats after tendon ligation injury (TL). EF_{50}, the von Frey filament force (g) that produces a 50% response, was a measure of mechanical sensitivity. Primary BMSCs were infused i.v. ($1.5x10^6$, or 1.5 M cells/0.2 ml) at 7d after TL. The EF_{50}s of BMSC-treated rats were increased after BMSC injection, indicating attenuation of hyperalgesia. **b.** At 6-17 d after BMSC, 6-β-naltrexol was administered i.v. There was a significant reduction of EF_{50} at 30 min after 20 mg/kg 6-β-naltrexol, indicating rekindling of hyperalgesia. Error bars are 95% confidence intervals of EF_{50}. ***, $p<0.001$, vs. Baseline and BMSC 6-17 d; *, $p<0.05$, vs. Veh and 4 mg/kg, n=4-8/Group. **c-f**. MOR immunostaining of RVM sections. Note increased fluorescence intensity and punctate in RVM neurons (Arrows) at 1w (**c**) and 8w (**e**) after BMSC infusion, compared to culture medium-treated (**d,f**) and naïve (**g**) controls. BMSC was infused at 7d after TL. **h**. Human BMSCs produced selective upregulation of MOR mRNA in RVM in rats after L5 spinal nerve ligation (SNL), a widely used rodent neuropathic pain model. BMSCs were infused at 7d after SNL and tissues were taken at 8w after BMSC infusion. Statistics: **a:** One-way ANOVA followed by post-hoc comparisons with Bonferroni corrections. **b**: Two-way ANOVA followed by post-hoc comparisons with Bonferroni corrections. **h**: Unpaired, Two-tailed Student's *t*-test.

Supplementary Fig. 2. BMSCs were isolated from GFP transgenic S-D rats, expanded in culture, and infused into the rat at 7d after TL. GFP-positive BMSCs (left column, green) were clearly accumulated in the lungs at 6h-1d after infusion and very few GFP cells were observed at 7d after infusion (*, alveolus; arrows: interalveolar septum). The DAPI counterstain (right) shows cell nuclei. Scale = 0.2 mm

Supplementary Fig. 3. RT-qPCR amplification plots confirming higher expression levels of 10 genes in PRI-BMSCs. Fluorescence intensity (ΔRn in log scale) is plotted against the PCR cycle number. Note leftward shift of the amplification curves for PRI-BMSCs (blue), compared to 20P-BMSCs (red) samples, indicating increased expression. GAPDH was an endogenous control. Fold increase in PRI cells is shown as PRI/20P ratio, n=3/gene.

Supplementary Fig. 4. Pretreatment with AMD3100 (plerixafor, 10 mg/kg/day, i.p. for 4 days, n=6; Veh, n=4), an antagonist of SDF-1 receptor CXCR4, did not affect BMSC-produced antihyperalgesia. P>0.05, Two-way ANOVA followed by post-hoc comparisons with Bonferroni corrections.

Supplementary Fig. 5. The effect of monocyte/macrophage depletion. **a**. Liposome-encapsulated clodronate (Lipo-Clo) was injected i.p. 3 times in three days (10 mg/2 ml/injection/rat). After the injections, peritoneal cells were collected and labeled with CD11b. Flow cytometry showed largely reduced CD-11b population (Right, lower panel). **b**. Reduction of macrophages was verified with CD68 (ED1)-immunostaining of spleen sections. Scale=0.2 mm.

Supplementary Fig. 6. **a**. Flow cytometry shows that 30.9% of gated population were positive for monocyte marker CD11b. This population was further purified by FACS. **b**. Monocytes show characteristic CD11b (upper) and CD68 (lower) immunostaining (green), counterstained with DAPI (blue). Scale = 0.05 μm.

Supplementary Fig. 7. The Rat Inflammatory Cytokines PCR Array of MC purified from PRI- or 20P-BMSC-treated rats (7d-TL). Genes with mean fold change (PRI/20P-treated) greater than ±2-fold are shown. Three genes, *Ccl2*, *Cxcl1* and Il1r1, showed higher expression levels in PRI

BMSC-treated MC (>2-fold) in all three samples (filled circles, arrows). Dashed lines indicate ±2-fold changes.

Supplementary Fig. 8. Injection of NVP CXCR2 20 (NVP) into the rostral dorsomedial medulla region did not affect BMSC-induced antihyperagesia. **a**. An image of brainstem section illustrating an example of the site of injection. Coronal brainstem sections were stained with Neurotrace^{TM} 500/525 Green fluorescent Nissal Stain (Invitrogen). Arrow indicates the injection needle track. The site of injection (circle) was located at the ventral end of medial longitudinal fasciculus (mlf) and the tectospinal tract (ts) field. **b**. NVP (200 pmol) was injected into the dorsomedial medulla region at 8w after BMSC infusion. The antihyperalgesic effect of BMSCs was maintained after NVP injection. The results of injecting vehicle into the RVM from Fig. 6b is plotted for comparison. One-way ANOVA followed by post-hoc comparisons with Bonferroni corrections.

Supplementary Fig. 9. Original western blot gel images for cropped images (rectangles) shown in Figures as indicated. Note that for Fig. 2g, the membranes were incubated with IRDye800CW-conjugated goat anti-rabbit IgG and IRDye680-conjugated goat anti-mouse IgG secondary antibodies (LI-COR) simultaneously and immunoreactivity for MOR and β-actin appeared on the same image.

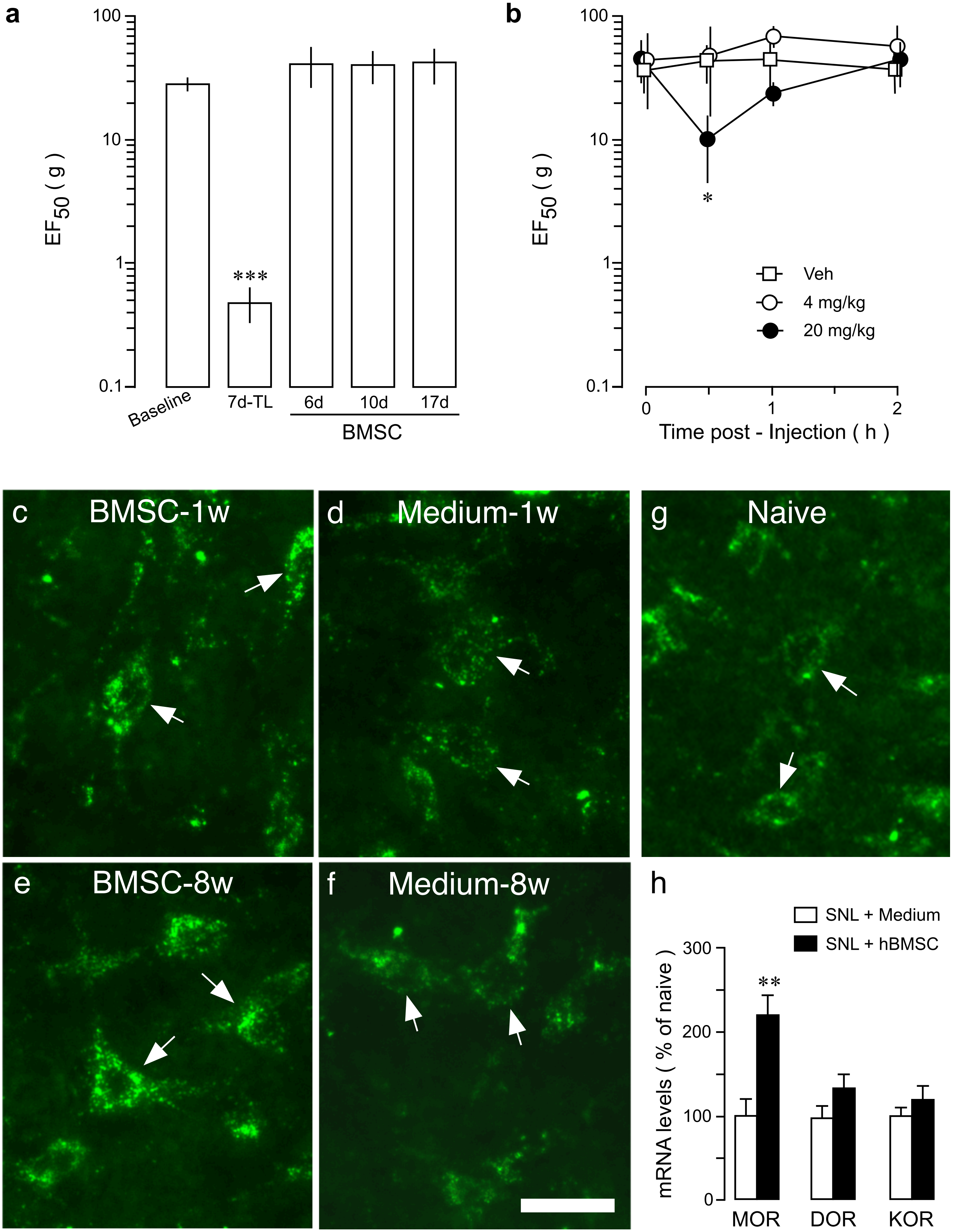

a
b
100
10
1
0.1
EF50 (g)
***
Baseline
7d-TL
6d
10d
17d
BMSC
*
Veh
4 mg/kg
20 mg/kg
0
1
2
Time post - Injection (h)
c
BMSC-1w
d
Medium-1w
g
Naive
e
BMSC-8w
f
Medium-8w
h
SNL + Medium
SNL + hBMSC
300
200
100
0
mRNA levels (% of naive)
**
MOR
DOR
KOR

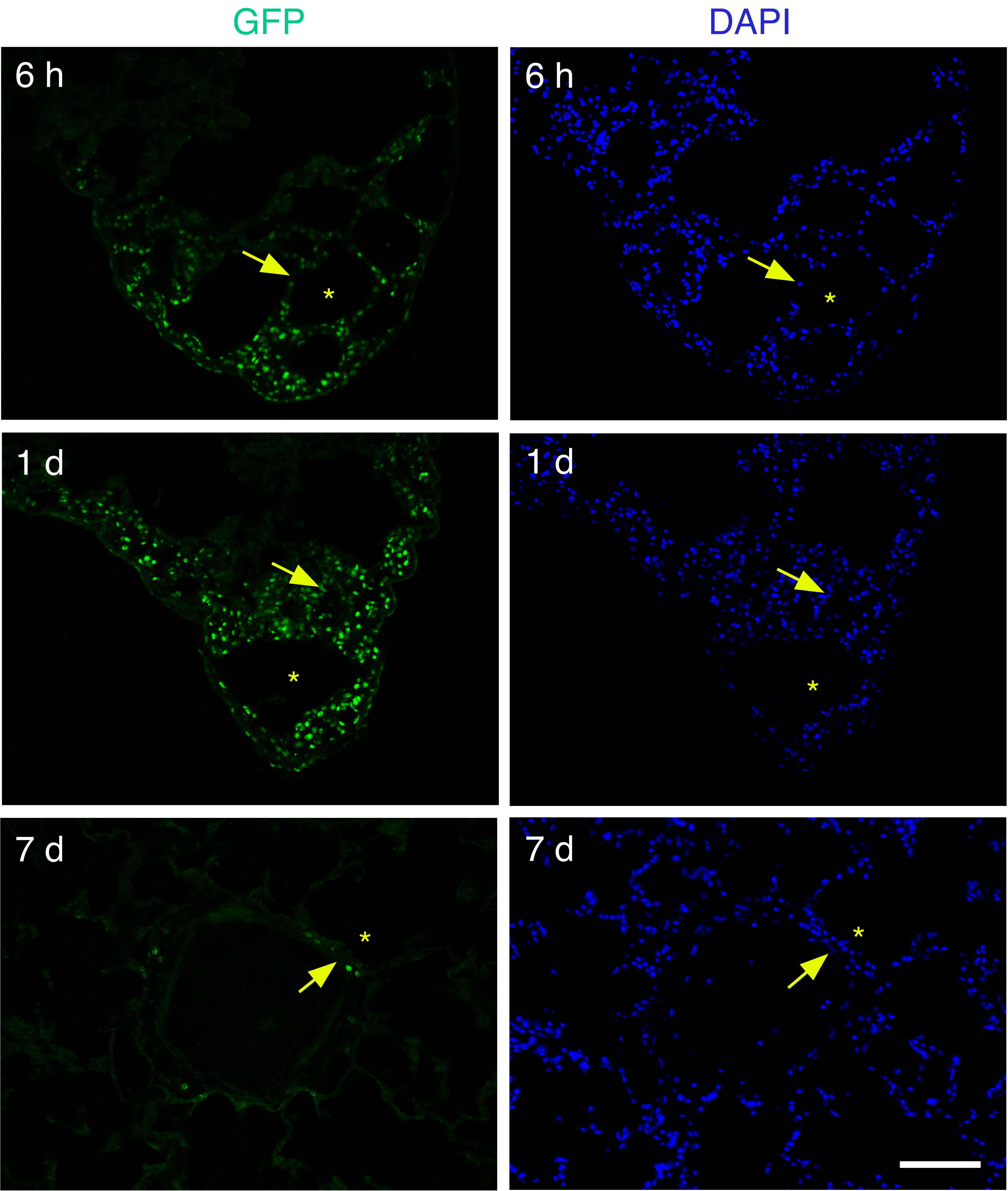

GFP
DAPI
6 h
6 h
1 d
1 d
7 d
7 d

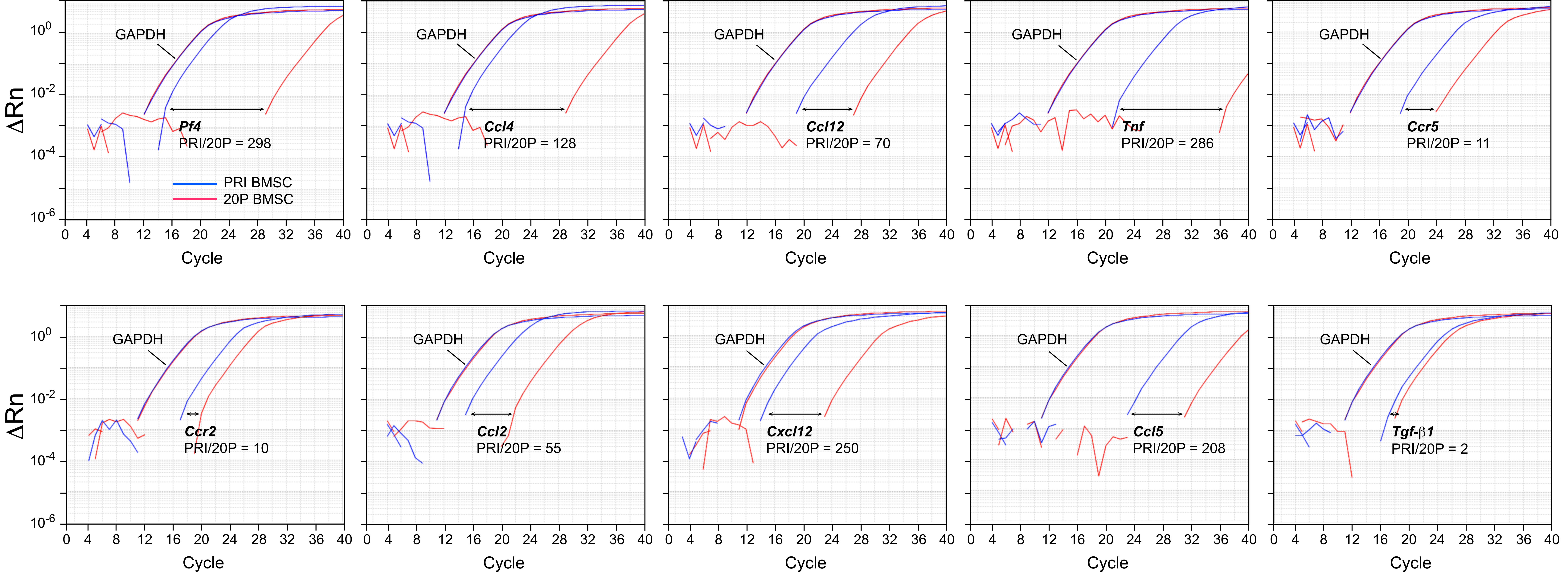

ΔRn
GAPDH
Pf4
PRI/20P = 298
PRI BMSC
20P BMSC
Ccl4
PRI/20P = 128
Ccl12
PRI/20P = 70
Tnf
PRI/20P = 286
Ccr5
PRI/20P = 11
Ccr2
PRI/20P = 10
Ccl2
PRI/20P = 55
Cxcl12
PRI/20P = 250
Ccl5
PRI/20P = 208
Tgf-β1
PRI/20P = 2
Cycle

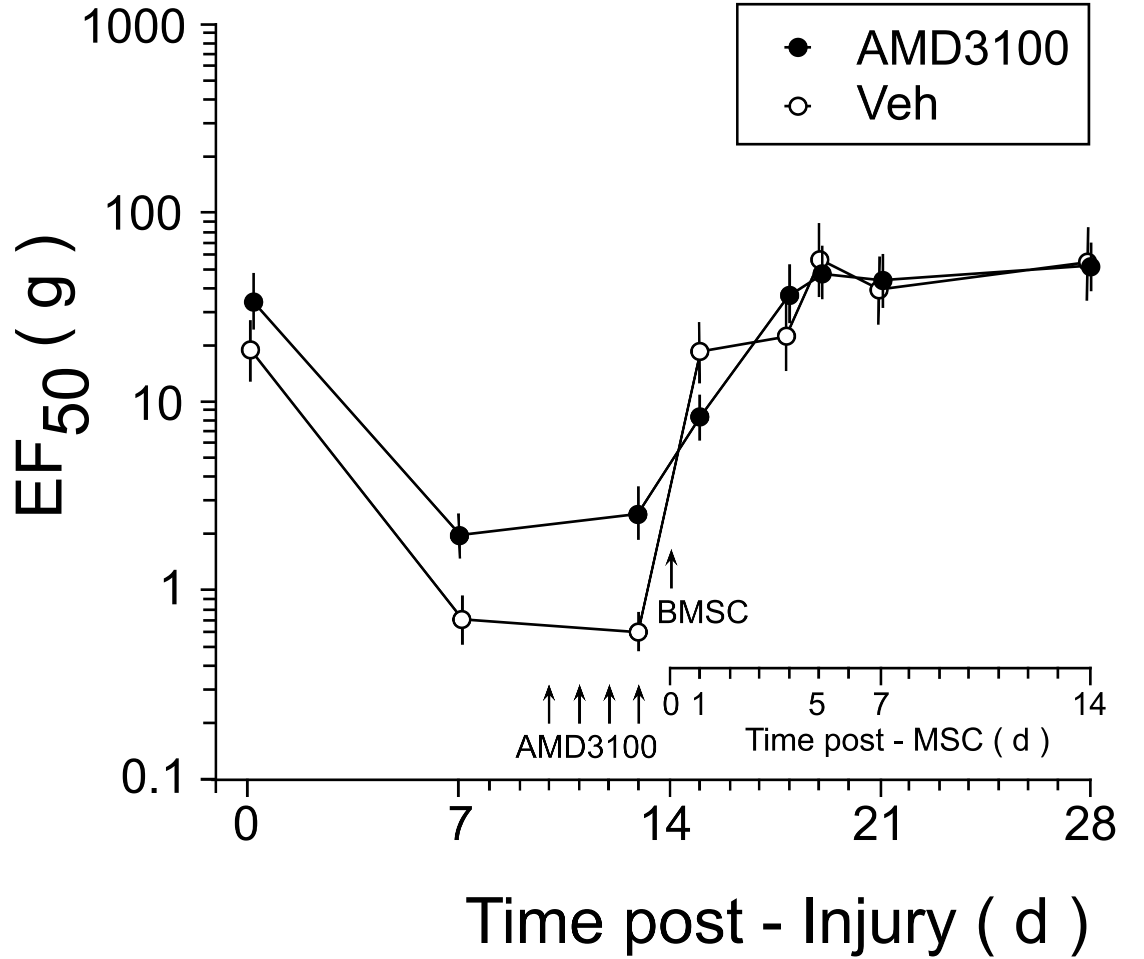

AMD3100
Veh
1000
100
10
1
0.1
EF50 (g)
BMSC
AMD3100
0 1 5 7 14
Time post - MSC (d)
0 7 14 21 28
Time post - Injury (d)

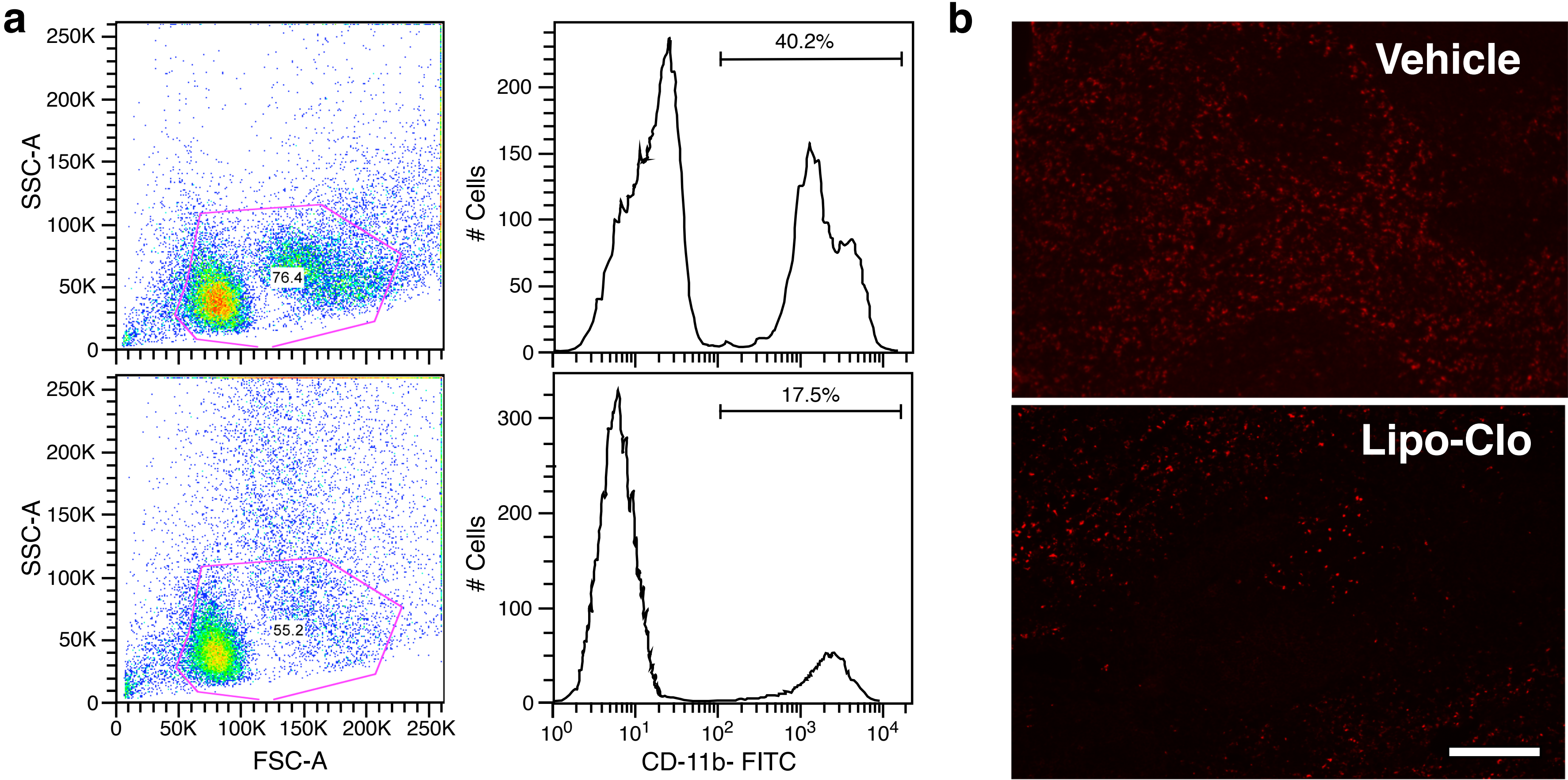
a
b
SSC-A
FSC-A
76.4
55.2
Cells
40.2%
17.5%
CD-11b- FITC
Vehicle
Lipo-Clo

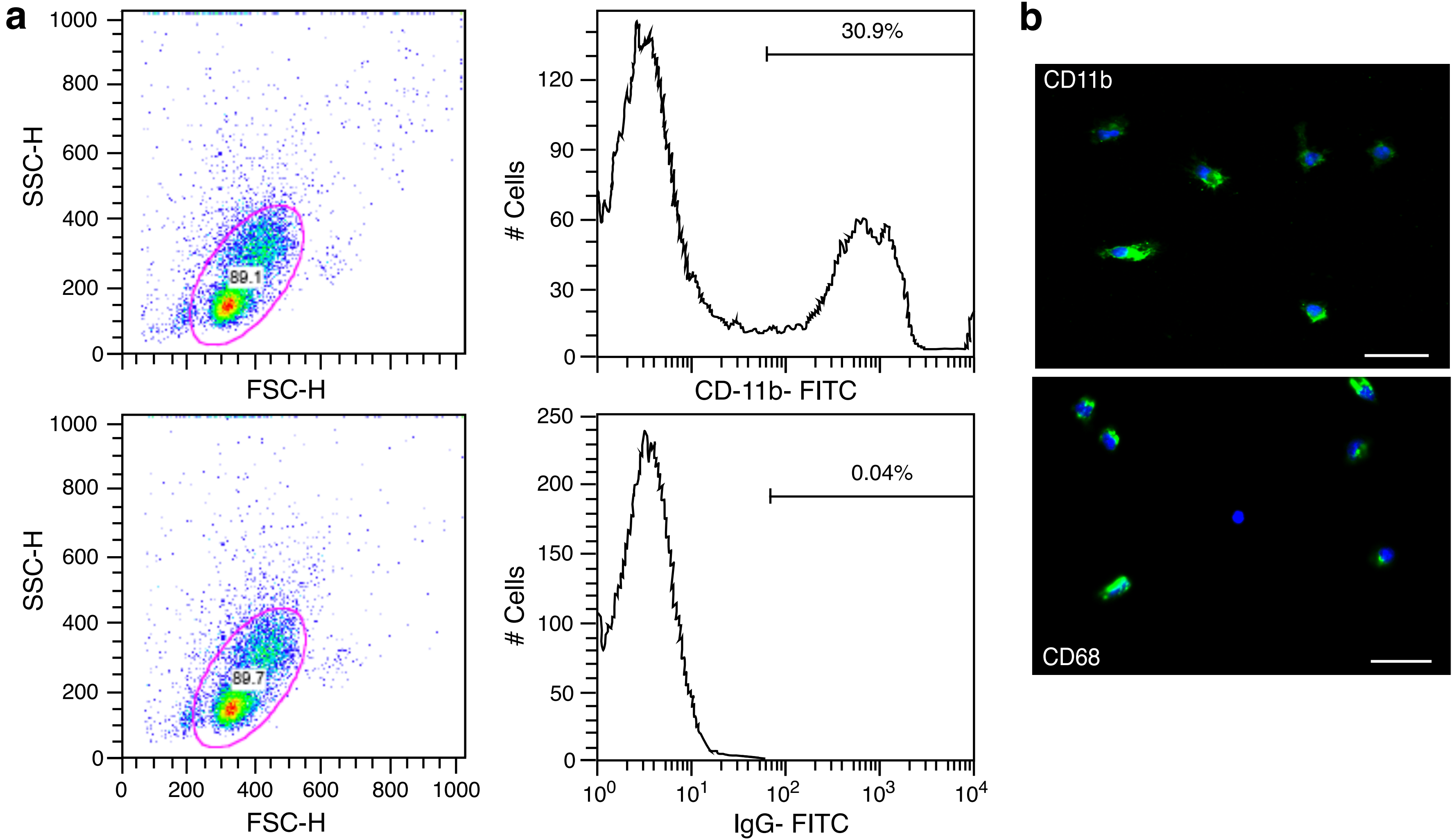
a
b
SSC-H
FSC-H
89.1
89.7
Cells
CD-11b- FITC
IgG- FITC
30.9%
0.04%
CD11b
CD68

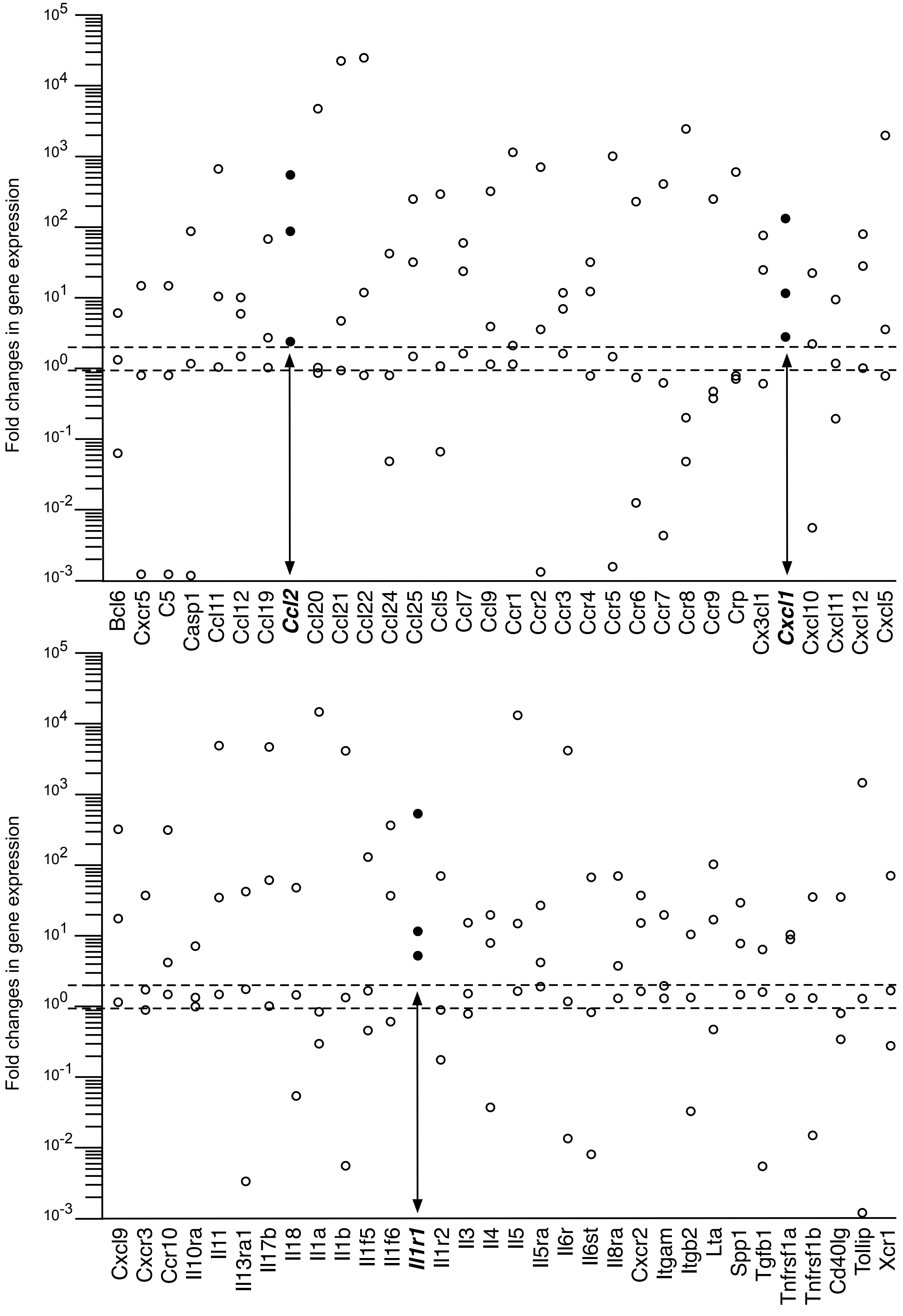

Fold changes in gene expression
10^5
10^4
10^3
10^2
10^1
10^0
10^-1
10^-2
10^-3
Bcl6 Cxcr5 C5 Casp1 Ccl11 Ccl12 Ccl19 Ccl2 Ccl20 Ccl21 Ccl22 Ccl24 Ccl25 Ccl5 Ccl7 Ccl9 Ccr1 Ccr2 Ccr3 Ccr4 Ccr5 Ccr6 Ccr7 Ccr8 Ccr9 Crp Cx3cl1 Cxcl1 Cxcl10 Cxcl11 Cxcl12 Cxcl5
Fold changes in gene expression
10^5
10^4
10^3
10^2
10^1
10^0
10^-1
10^-2
10^-3
Cxcl9 Cxcr3 Ccr10 Il10ra Il11 Il13ra1 Il17b Il18 Il1a Il1b Il1f5 Il1f6 Il1r1 Il1r2 Il3 Il4 Il5 Il5ra Il6r Il6st Il8ra Cxcr2 Itgam Itgb2 Lta Spp1 Tgfb1 Tnfrsf1a Tnfrsf1b Cd40lg Tollip Xcr1

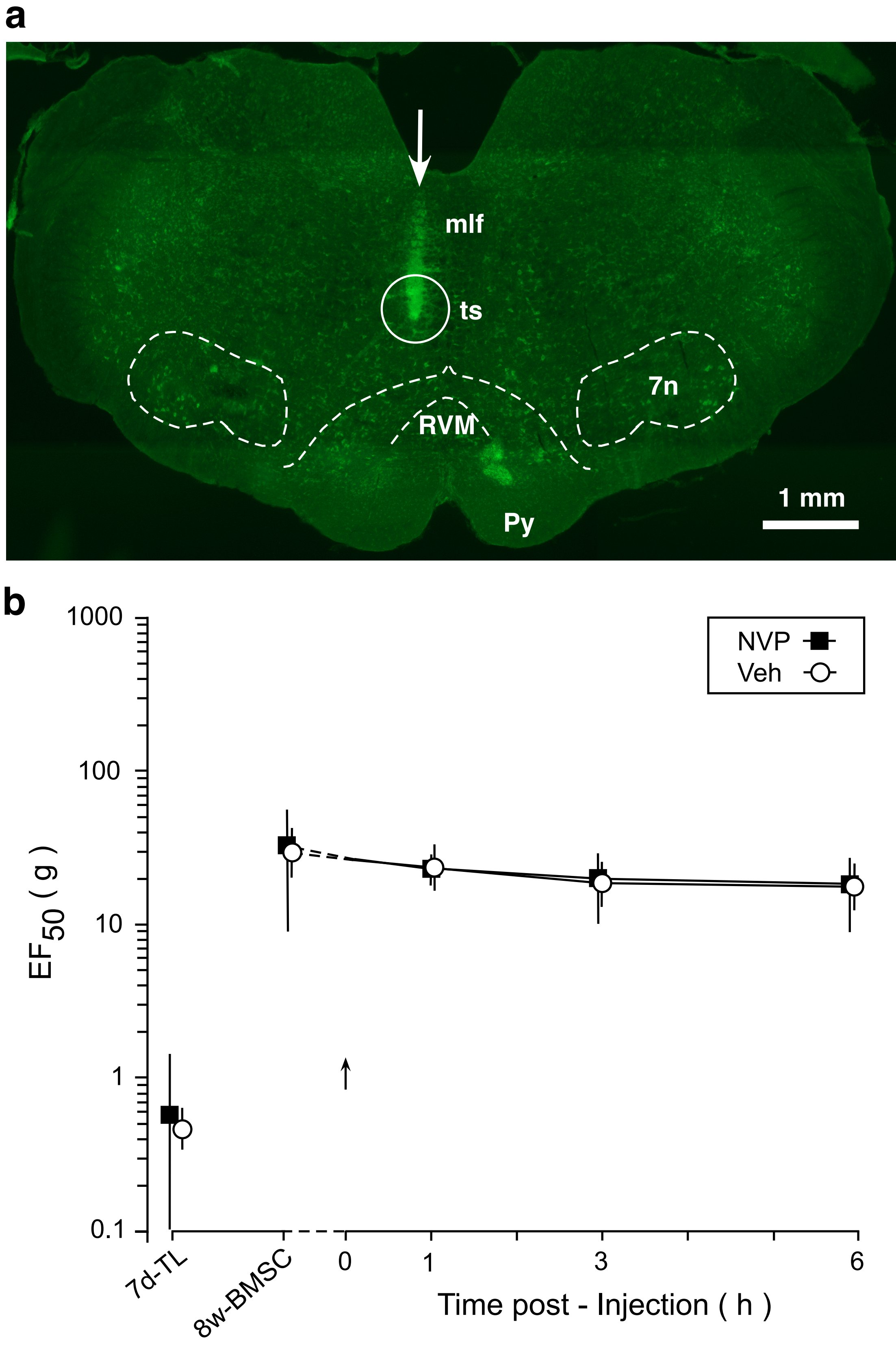
a
mlf
ts
7n
RVM
Py
1 mm
b
NVP
Veh
1000
100
10
1
0.1
EF50 (g)
7d-TL
8w-BMSC
0
1
3
6
Time post - Injection (h)

Fig. 1C.

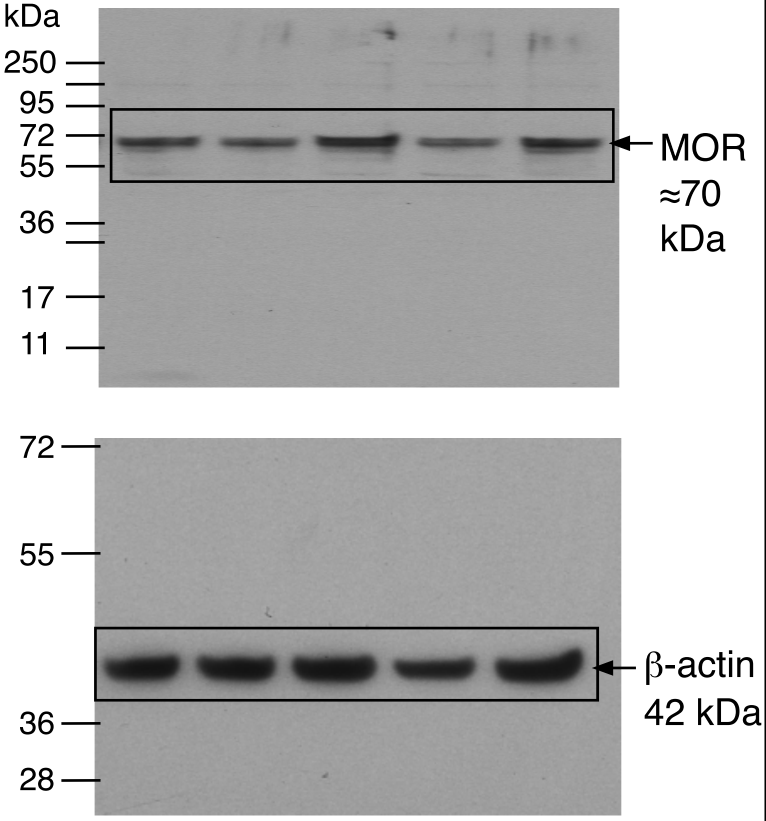

Fig. 2a.

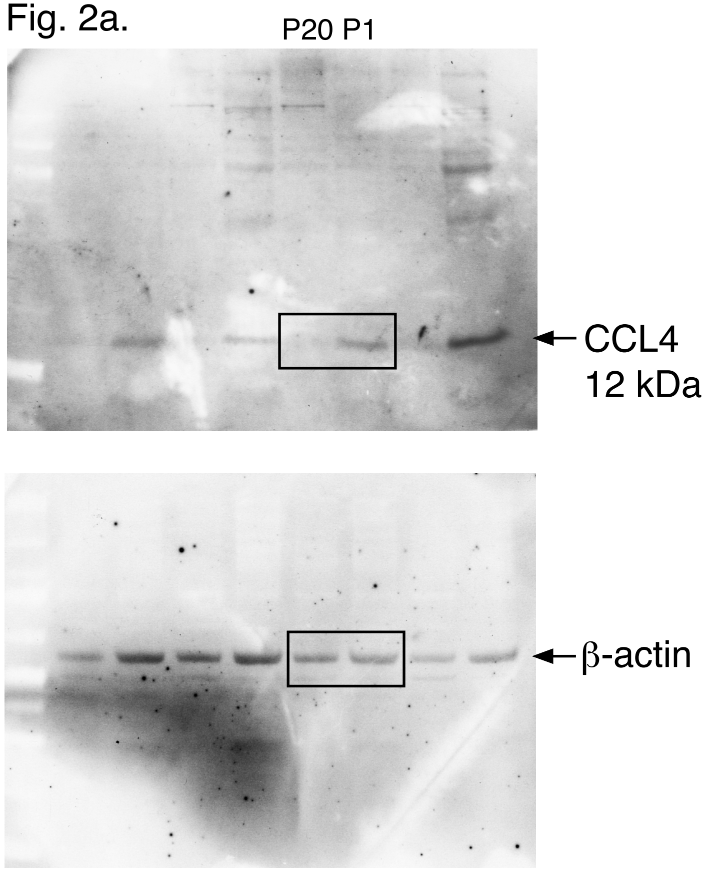

Fig. 2e.

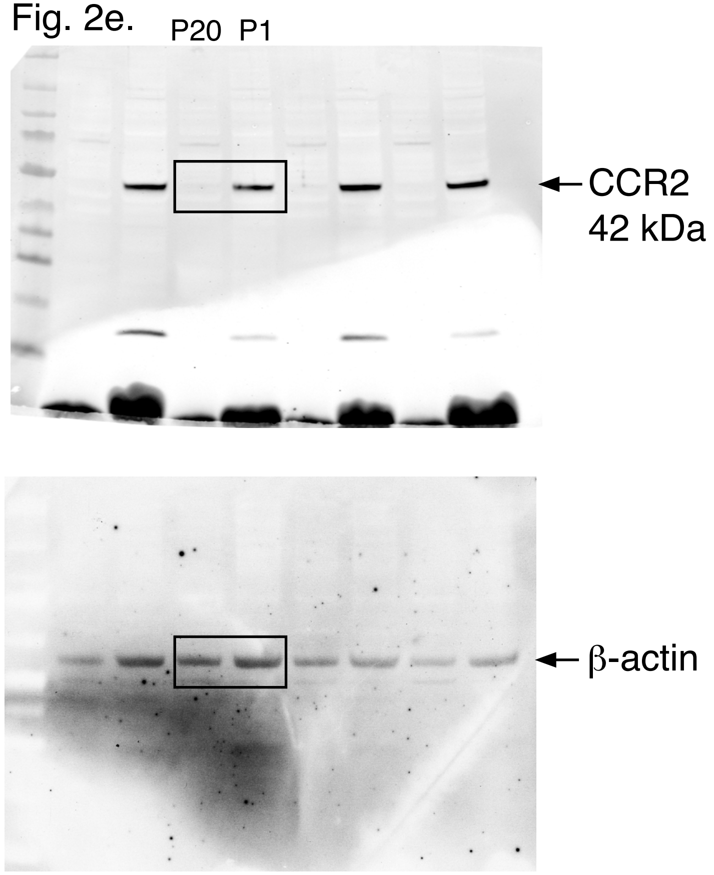

Fig. 2g.

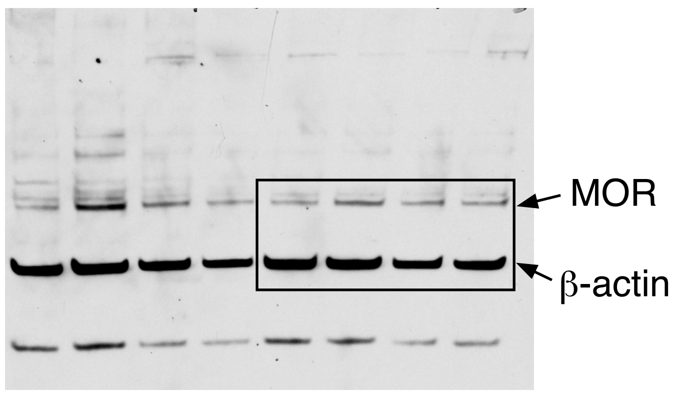

Fig. 5e.

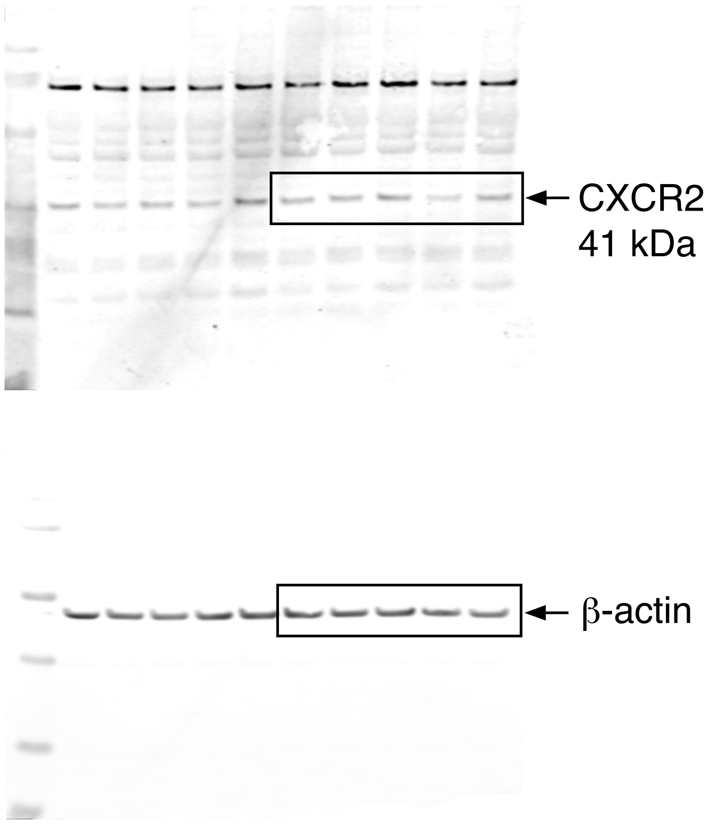

Fig. 6e.

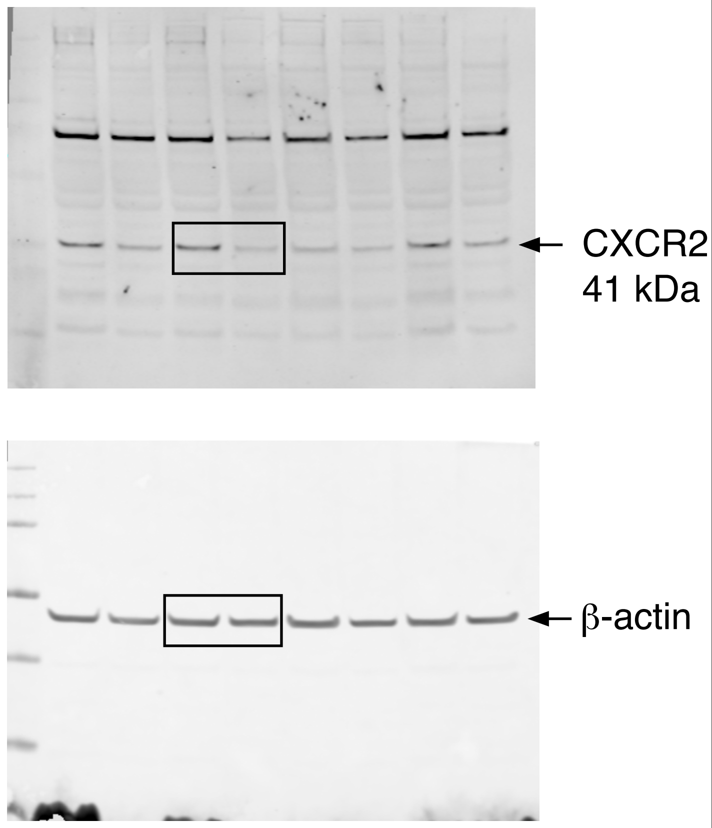

Supplement: Supplementary file 1 — Supplementary materials [file 41598_2017_10251_MOESM1_ESM.pdf]
